# An investigation of methods to enhance adhesion of conductive layer and dielectric substrate for additive manufacturing of electronics

**DOI:** 10.1038/s41598-024-61327-5

**Published:** 2024-05-06

**Authors:** Zhiguang Xu, Jizhuang Hui, Jingxiang Lv, Dongjie Wei, Zhiqiang Yan, Hao Zhang, Junjie Wang

**Affiliations:** Key Laboratory of Road Construction Technology and Equipment of MOE, Chang’an University, Xi’an, China

**Keywords:** Additive manufacturing, Inkjet printing, PEEK, Nanoparticle silver ink, Surface modification, Binding force, Mechanical engineering, Characterization and analytical techniques

## Abstract

Additive manufacturing of conductive layers on a dielectric substrate has garnered significant interest due to its promise to produce printed electronics efficiently and its capability to print on curved substrates. A considerable challenge encountered is the conductive layer’s potential peeling due to inadequate adhesion with the dielectric substrate, which compromises the durability and functionality of the electronics. This study strives to facilitate the binding force through dielectric substrate surface modification using concentrated sulfuric acid and ultraviolet (UV) laser treatment. First, polyetheretherketone (PEEK) and nanoparticle silver ink were employed as the studied material. Second, the surface treatment of PEEK substrates was conducted across six levels of sulfuric acid exposure time and eight levels of UV laser scanning velocity. Then, responses such as surface morphology, roughness, elemental composition, chemical bonding characteristics, water contact angle, and surface free energy (SFE) were assessed to understand the effects of these treatments. Finally, the nanoparticle silver ink layer was deposited on the PEEK surface, and the adhesion force measured using a pull-off adhesion tester. Results unveiled a binding force of 0.37 MPa on unmodified surface, which escalated to 1.99 MPa with sulfuric acid treatment and 2.21 MPa with UV laser treatment. Additionally, cross-approach treatment investigations revealed that application sequence significantly impacts results, increasing binding force to 2.77 MPa. The analysis further delves into the influence mechanism of the surface modification on the binding force, elucidating that UV laser and sulfuric acid surface treatment methods hold substantial promise for enhancing the binding force between heterogeneous materials in the additive manufacturing of electronics.

## Introduction

**Figure 1:**
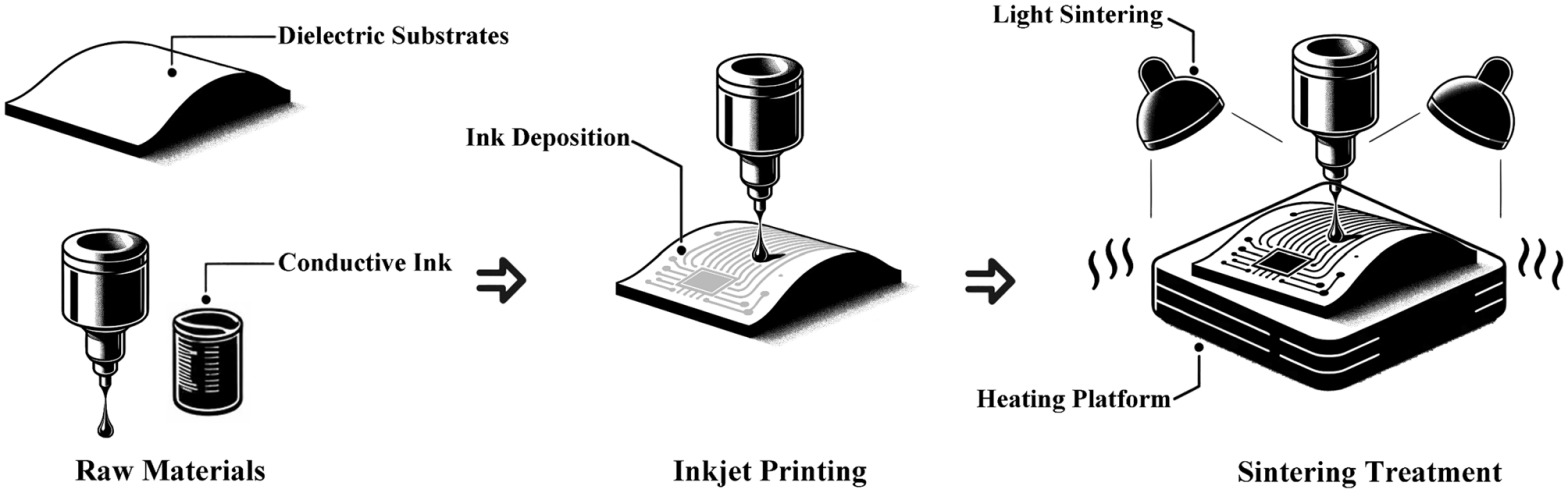
Schematic representation of 3D inkjet printing fabrication.

The advent of additive manufacturing has ushered in a new era of possibilities in creating conductive layers on dielectric substrates. This advancement is promising for the economical and efficient production of printed electronics, with the extra advantage of facilitating printing on curved substrates^1,2^. Driving this innovation, technologies such as aerosol jet printing^3^, laser direct writing^4^, screen printing^5^ and inkjet printing^6^ offer varied approaches to creating conductive layers on dielectric substrates. Inkjet printing technology is precise in depositing minuscule ink droplets, enabling high-resolution conductive patterns on flat and curved surfaces. This method combines efficiency, versatility, and cost-effectiveness, making it particularly suited for advancing the production of complex printed electronics. As demonstrated in Fig. 1, inkjet printing technology facilitates the creation of conductive layers with high resolution and accuracy through precise ink droplet deposition. This process entails precisely ejecting minuscule droplets of conductive ink onto a substrate, followed by a sintering treatment that transforms the deposited ink into a conductive layer^7,8^. Such advancements have enabled the manufacturing of a variety of innovative products, including 3D antennas^9^, sensors^10^, flexible electronics^11^, and conformal circuits^12^. This technology simplifies the fabrication process and provides various benefits such as cost-effectiveness, rapid prototyping, and customization, which are pivotal for advancing the frontier of electronics manufacturing. However, a significant hurdle emerges from the potential peeling of the conductive layer due to inadequate adhesion with the dielectric substrate, which threatens the durability and operational efficacy of the electronics^13^.

The pathway to overcoming the adhesion challenge hinges on the meticulous selection of surface modification techniques tailored to the distinct characteristics of different substrates^14^. By precisely aligning the modification technique with the substrate’s inherent properties, a conducive environment for enhanced adhesion with other layers can be fostered. Amidst the spectrum of substrates, PEEK has garnered attention due to its unique attributes. PEEK, a high-performance thermoplastic, is revered for its superior mechanical properties, chemical resilience, and biocompatibility, making it a substrate of choice across many industries, including aerospace and biomedical sectors^15,16^. Moreover, PEEK material can be manufactured utilizing fused deposition modeling (FDM) technology, further amplifying its allure in modern industrial applications^17,18^. It allows for the design of different substrate structures to create conformal circuits, leveraging the technology’s ability to deposit material layer by layer to build complex geometries^19^. Despite its favorable attributes, PEEK’s inherent hydrophobicity and low SFE pose a substantial challenge in achieving robust adhesion with conductive inks^20^. Specifically, PEEK’s low SFE and inherent hydrophobic characteristics impede its ability to form solid and consistent bonds with materials like conductive inks. Furthermore, additional surface roughness is introduced when PEEK is 3D printed using the FDM technique^21^, thereby challenging the achievement of a reliable interface between PEEK and subsequent coatings or layers.

**Figure 2:**
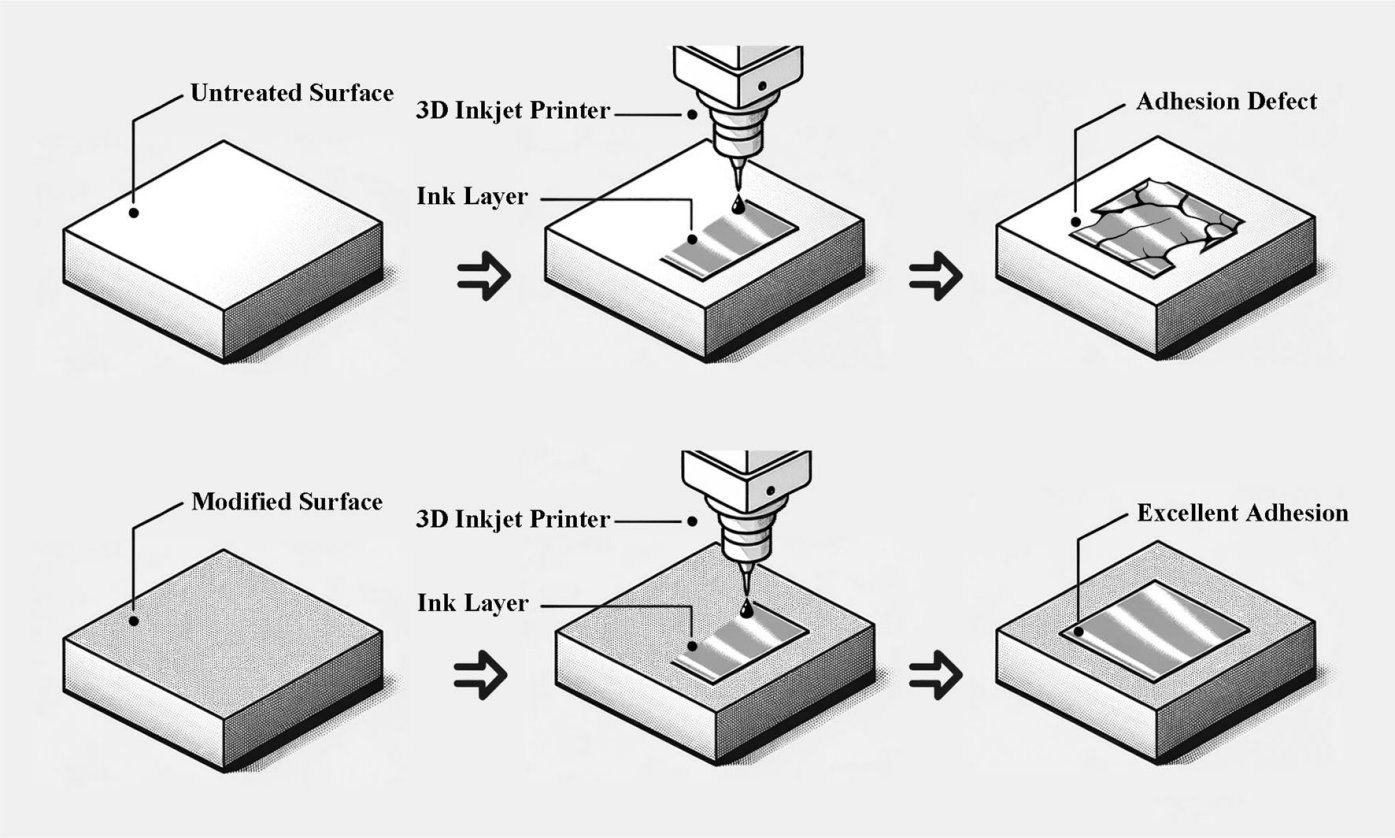
Schematic representation of adhesion enhancement through surface modification.

The quest for enhanced adhesion between PEEK materials and subsequent coatings has led researchers to explore many surface treatment techniques. Some prevalent methods include plasma treatment^22^, chemical etching^23^, mechanical abrasion^24^, and laser treatment^25^. These methods aim to alter PEEK’s surface chemistry or topography to foster better adhesion with coatings. Among these, concentrated sulfuric acid has been recognized as an effective treatment method to modify the surface properties of various materials, enhancing their wettability, adhesion, and overall performance. Sulfonation reactions can lead to changes in the microstructure of PEEK materials, especially the formation of porous networks. Longer sulfonation time usually leads to more obvious porous structures. Still, there is an optimal duration to balance porosity and material integrity, and excessive sulfonation may lead to the degradation of PEEK materials^26,27^. The SFE of PEEK is significantly enhanced by sulfuric acid treatment, fostering better wettability and ensuring a more uniform spread of resin composite cement, demonstrating notable improvements in bonding strength^28^. Another study highlighted that etching with 98% sulfuric acid significantly enhanced the bond strength of certain adhesives to PEEK composite materials, compared to other treatment methods such as hydrofluoric acid or sandblasting, which did not establish any adhesion^23^. Such enhancements are crucial, especially for robust adhesion between PEEK and conductive ink layers. Concentrated sulfuric acid treatments induce chemical alterations on the PEEK surface, introducing functional groups that can further improve adhesion and compatibility with various coatings^29,30^. While many of these studies are oriented toward medical applications and emphasize enhanced biocompatibility, the consensus underscores sulfuric acid’s versatility and effectiveness for PEEK surface modification^31,32^. Transitioning from chemical to physical modification techniques, the advent of UV laser treatment presents an alternate yet equally promising avenue in surface engineering. UV lasers do not induce a thermal effect, which is beneficial in preserving the integrity of materials during the surface treatment process^33^. Studies on PEEK and its derivatives have underscored the potential of UV laser treatments in sculpting intricate surface textures, be it grooves, sub-micron structures, or other bespoke patterns^34^. These topographical augmentations facilitate enhanced mechanical interlocking and bolstering adhesion with applied layers like metal coatings or resin-based materials^35,36^. Beyond mere surface structuring, UV lasers drive chemical transformations at the substrate’s surface. Such treatments, especially on PEEK, introduce specific functional groups, elevating the material’s compatibility and adhesive potential with various coatings^36,37^. This chemically enhanced adhesion proves indispensable in applications demanding robust bonding, like the integration of conductive ink layers. Moreover, UV laser treatments offer a unique avenue to modulate wettability. By tailoring surface textures, materials can be customized to exhibit desired hydrophilic or hydrophobic characteristics, a trait crucial for applications demanding precise surface–liquid interactions^38,39^. The discourse in the literature predominantly orbits around medical applications^31,40^, showing a discernible lacuna focusing on improving the conductive layer bond strength for other industrial applications. This gap underscores a rich avenue for future research, particularly in electronics, aerospace, and other high-performance applications. Establishing a solid bond between PEEK materials and conductive layers would pave the way for advanced electronic applications. As demonstrated in Fig. 2, 3D inkjet printing technology for depositing a conductive ink layer on the modified surface achieves a superior bonding effect, circumventing detrimental issues such as delamination and cracking engendered by inadequate bonding adherence. Through a rigorous comparative analysis of sulfuric acid and UV laser treatment, this study aspires to delineate a roadmap that navigates the intricacies of surface modifications, elucidating the path towards achieving optimal adhesion between the PEEK and the conductive layer.

This investigation delves into the nuanced interface between PEEK and conductive ink layers, aiming to optimize the binding force at this juncture. The study systematically explores surface modification techniques, notably concentrated sulfuric acid treatment and UV laser treatment, to discern their effects on PEEK substrates. It extends to modulating durations in sulfuric acid treatment and varying application speeds in UV laser treatment to understand how these modalities alter surface properties and impact adhesion with conductive ink layers. Additionally, the study investigates the effects of combining these treatments sequentially, assessing how the order of application influences the overall adhesion performance and surface characteristics. Firstly, the study examines the alterations in surface morphology resulting from concentrated sulfuric acid and UV laser treatments. Secondly, it investigates the chemical transformations induced by these treatments. Thirdly, the research explores the variations in surface wettability and SFE post-treatment. Fourthly, a quantitative assessment of the surface binding force is undertaken, establishing a correlation between the binding force and the observed morphological and chemical alterations. Lastly, the study expands to include cross-approach treatments, wherein the combined effects of sulfuric acid and UV laser treatments in varying sequences are examined to ascertain their potential to enhance the binding force further. Through a meticulous examination, this study aspires to lay a foundation for enhanced adhesion in 3D printing applications, ensuring superior binding performance with nanoparticle conductive ink layers on PEEK substrates, which holds promise for advancing the frontier of additive manufacturing.

## Materials and methods

### Materials and suppliers

The PEEK filament, with a diameter of 1.75 mm, was sourced from INTAMSYS Technology Co., Ltd (Shanghai, China). The filament exhibited the following properties: a density of 1.3 g/cm^3^, tensile strength of 99.9 MPa, Young’s modulus of 3738 MPa, flexural strength of 147 MPa, and a flexural modulus of 3612 MPa. Additionally, concentrated sulfuric acid (H_2_SO_4_, 95–98%), ethanol (C_2_H_6_O), and acetone (C_3_H_6_O) were procured from Fuyu Chemical Co., Ltd (Shandong, China). Diiodomethane was obtained from Zhanyun Chemical Co., Ltd (Shanghai, China).

### Fabrication of PEEK samples

PEEK specimens were fabricated using an FDM 3D printer (FUNMAT PRO 410, INTAMSYS Technology Co., Ltd., Shanghai, China), with the following settings: printing temperature of 440 °C, build plate temperature of 130 °C, and chamber temperature of 90 °C. The layer height was set to 0.15 mm, utilizing a 100% infill density with a line pattern at a print speed of 60 mm/s. The specimens were designed with dimensions of 50 mm × 50 mm × 4 mm.

### Surface refinement of PEEK samples

Before surface modification, the 3D printed PEEK samples were first subjected to a foundational polishing process. This initial step was deemed essential due to the inherent roughness imparted by the 3D printing method. This preparatory stage involved sanding the samples sequentially using silicon carbide (SiC) sandpaper, starting from 240 and advancing to 3000 mesh, ensuring a consistent surface roughness across the specimen. This finalized product, termed Sandpaper-Polished PEEK (SP-PEEK), served as the reference sample for subsequent modifications.

### Sulfonation treatment

The SP-PEEK specimens underwent a sulfonation process wherein they were submerged in concentrated sulfuric acid at ambient temperature for varying time intervals: 30 s (SPEEK30), 60 s (SPEEK60), 120 s (SPEEK120), 180 s (SPEEK180), 240 s (SPEEK240), and 300 s (SPEEK300). Once the stipulated time elapsed, the samples were promptly shifted into deionized water to terminate the reaction.

### Ultraviolet laser treatment

The PEEK samples were exposed to a UV laser process, utilizing a laser marking machine (SEAL-355-10S, JPT Opto-electronics Co., Ltd., Shenzhen, China) equipped with a 355 nm UV pulsed laser system. Diverse microstructures with distinct morphologies were crafted by modulating the laser’s scanning speed. Speeds were set at 2700 mm/s (UV-PEEK27), 2400 mm/s (UV-PEEK24), 2100 mm/s (UV-PEEK21), 1800 mm/s (UV-PEEK18), 1500 mm/s (UV-PEEK15), 1200 mm/s (UV-PEEK12), 900 mm/s (UV-PEEK9), and 600 mm/s (UV-PEEK6). While adjusting the scanning speed, other processing parameters remained consistent: scanning was conducted once, with a line spacing of 0.05 mm, an electric current of 1 amp, and a frequency set at 30 kHz. The operational distance of the laser was maintained at 14 cm.

### Post-treatment cleaning and preservation protocol

After each modification—sanding, sulfonation, or UV laser treatment—the PEEK samples underwent a uniform cleaning process. This involved an ultrasonic bath in acetone, ethanol, and deionized water, each for 10 min, to remove any surface contaminants. The samples were then dried under a vacuum at 70 °C and hermetically sealed to maintain their pristine state before further testing or analysis. This rigorous protocol ensured that all PEEK specimens were comparably clean and preserved, allowing for an accurate assessment of the effects of each treatment type.

### Characterization of materials

#### Surface morphology examination

A white light interferometer (Atometrics-NA500, Boardstone Intelligent Co. Ltd., Shenzhen, China) was employed to assess the samples’ three-dimensional topography and surface roughness. The roughness test was carried out in five distinct areas on each sample surface, measuring 0.919 mm × 0.575 mm. The average value from these regions was computed to represent the sample roughness, denoted as the arithmetic mean height of the surface (Sa).

The surface microstructure of the samples was analyzed using a field emission scanning electron microscope (GeminiSEM 360, Carl Zeiss Co., Ltd., Oberkochen, Germany). Before the observation under the microscope, the samples were coated with a conductive coating of gold (Au) using a Mini Coater from Supro Instruments Co., Ltd. (Shenzhen, China). The established parameters for this coating were a power setting of 7W, a coating time of 180 s, and an operating pressure of 5 Pa.

#### X-ray photoelectron spectroscopy examination

An X-ray photoelectron spectrometer (XPS, PHI 5000 VersaProbe III, ULVAC-PHI Inc., Chigasaki, Japan) was harnessed to scrutinize the surface composition of PEEK samples. The examination prominently centered on quantifying the relative abundances of carbon (C), oxygen (O), and sulfur (S) on the samples’ surfaces. Further depth was added by analyzing the functional groups’ content, varieties, and relative ratios detected in the C1s peak spectrum. Notably, the carbon–carbon (C–C) spectral peak, pinpointed at 284.8 eV, was consistently used as a reference anchor for charge correction throughout the analysis.

#### Contact angle examination

The wettability of the sample surface was examined using a contact angle measuring instrument (SDC-350, ShengDing Precision Instrument Co., Ltd, China). The sessile drop method was employed, and measurements were taken at five distinct locations on the surface of each sample. A droplet volume of 2 μL was used for each measurement. The static contact angle was then determined by averaging the obtained values.

#### Surface free energy analysis

The material's SFE is a crucial property influencing various phenomena, including wetting, adhesion, and phase transition. The Owens, Wendt, Rabel, and Kaelble (OWRK) methods were used to estimate SFE on solid surfaces through contact Angle measurements and different test liquids^41,42^.

The Young’s equation which describes the balance of interfacial tensions (or surface energies) at the three-phase boundary (solid-liquid-gas) and can be represented as in Eq. (1):

1 $$\gamma_{s} = { }\gamma_{sl} + { }\gamma_{l} {\text{ cos}}\theta ,$$

where ${\gamma }_{s}$ is the interfacial tension between solid and gas (solid SFE), ${\gamma }_{sl}$ is the interfacial tension between solid and liquid, ${\gamma }_{l}$ is the surface tension of the liquid (in contact with gas), and θ is the contact angle.

The OWRK method extended the Young’s equation by decomposing the surface energies into dispersive and polar components. Now, considering that the SFE of both solid and liquid can be split into two components, dispersive (d) and polar (p). ${\gamma }_{sl}$ can be represented as:

2 $$\gamma_{sl} = \gamma_{s} + { }\gamma_{l} - { }2\left( {\gamma_{s}^{d} \gamma_{l}^{d} } \right)^{0.5} - { }2\left( {\gamma_{s}^{p} \gamma_{l}^{p} } \right)^{0.5} ,$$

where ${\gamma }_{s}^{d}$ is the solid dispersive component, ${\gamma }_{s}^{p}$ is the solid polar component, ${\gamma }_{l}^{d}$ is the liquid dispersive component and ${\gamma }_{l}^{p}$ is the liquid polar component. Combining Young’s equation:

3 $$\gamma_{l} \left( {1 + {\text{cos}}\theta } \right) = { }2\left( {\gamma_{s}^{d} \gamma_{l}^{d} } \right)^{0.5} + { }2\left( {\gamma_{s}^{p} \gamma_{l}^{p} } \right)^{0.5}$$

Water and diiodomethane were chosen for the SFE measurement. Typically, water has a dispersive component of surface tension (${\gamma }_{l}^{d}$) at 21.8 mJ/m^2^, a polar component (${\gamma }_{l}^{p}$) at 51.0 mJ/m^2^, and a total surface tension (${\gamma }_{l}$) of 72.8 mJ/m^2^. Diiodomethane’s surface tension components include a dispersive component of 50.8 mJ/m^2^ and a polar component of 2.3 mJ/m^2^, culminating in a total surface tension of 53.1 mJ/m^2^.

### Preparation of nanoparticle silver ink layer

**Figure 3:**
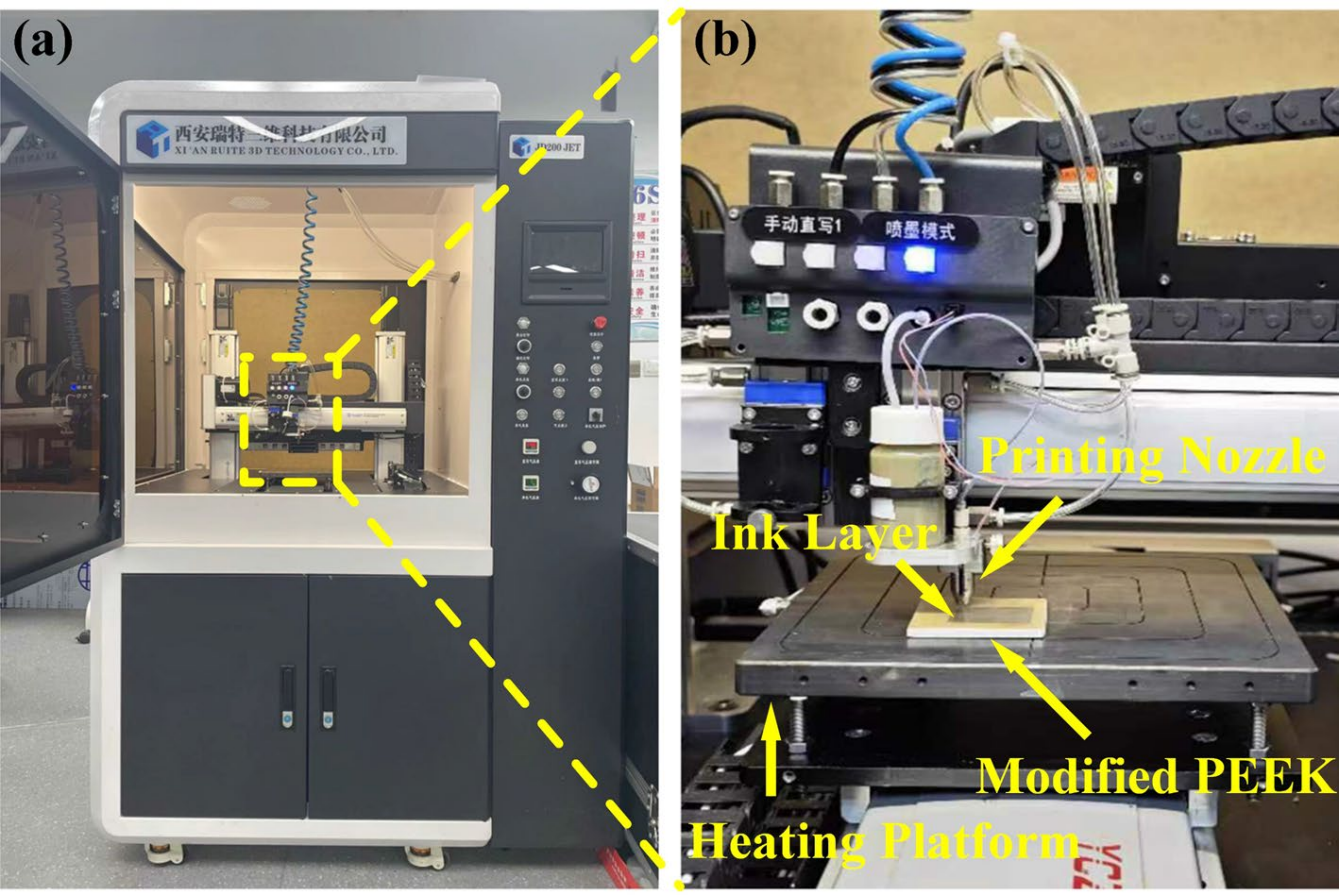
Inkjet 3D printing: (**a**) equipment; (**b**) operation process.

To layering silver ink on PEEK, nanoparticle silver ink (BroadCON-INK550, BroadTeko Technology Co., Ltd., Beijing, China) was selected. This ink comprised nanoparticles with diameters ranging from 30–50 nm. Its viscosity was 5–6 cp, with a silver content of 25–30 wt%. The ink exhibited a square resistance of 1–2 mΩ/□/mil. The piezoelectric inkjet 3D printing equipment (JD200 Jet, Ruite 3D Technology Co., Ltd., Xi’an, China) as shown in Fig. 3 a, was employed for precisely depositing this ink on the modified PEEK material. Operating on a drop-on-demand mechanism, the machine printed a silver layer measuring 30 × 30 mm^2^ with a thickness of 2 μm onto the treated PEEK substrate. With an aperture of 60 μm, the printing nozzle functioned at an operating pressure of 0.4 MPa. While printing, the equipment maintained a steady speed of 10 mm/s, rhythmically ejecting droplets at 150 Hz; the platform was heated to 100 ℃ throughout the process. The operation process is depicted in Fig. 3b. Upon completion of the printing process, the ink layers were subjected to sintering to enhance their structural and electrical integrity. This was achieved using a custom-designed near-infrared sintering apparatus operating in a flicker radiation mode. The PEEK samples with the ink layer were stationed 4 cm from the radiation source. Each sample experienced a rapid 3 s sintering exposure, a process that was repeated five times to guarantee thorough sintering.

### Binding force examination

**Figure 4:**
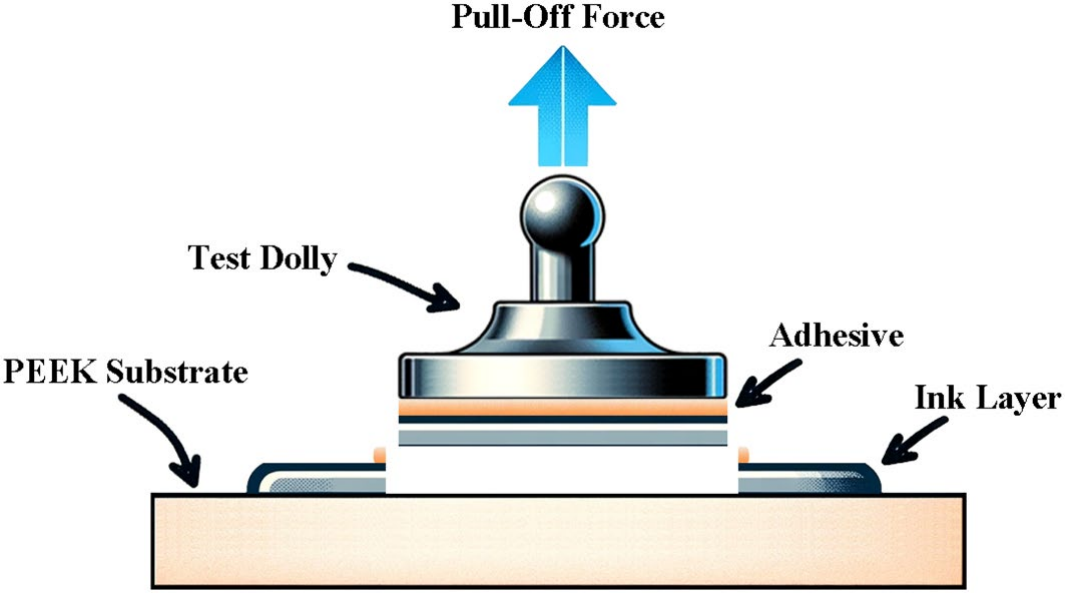
Schematic representation of binding force examination.

The binding force of the nanoparticle silver ink layer on the PEEK substrate was determined using an automatic digital pull-off adhesion tester (BGD 500/S, Biuged Precise Instruments Co., Ltd., Guangzhou, China). This device offered measurements with an accuracy of 0.01 MPa. The pressurization rate during the tests was maintained at 0.1 MPa/s. The procedure involved attaching a test dolly, with a diameter of 20 mm, to the sample surface. This was achieved using a high-performance adhesive, specifically the 3 M™ Scotch-Weld™ DP 420 epoxy resin. Post application, the assembly was left undisturbed at ambient conditions for 24 h, allowing the adhesive bond to mature. After this curing period, a dolly cutter was used to incise around the dolly’s perimeter, ensuring the cut penetrated the foundational PEEK layer. The adhesion tester then exerted a pull force on the dolly, measuring the strength required to detach the silver layer from the substrate. The schematic representation of the test process is shown in Fig. 4. Each sample group underwent this testing procedure thrice. The results from these repetitions were then averaged, ensuring a comprehensive and reliable representation of the binding force between the silver ink and the PEEK substrate.

## Results and discussion

The systematic exploration undertaken in this study aimed to shed light on the surface modification of 3D printed PEEK materials using two distinct methodologies: concentrated sulfuric acid treatment and UV laser treatment.

### Surface morphology

**Figure 5:**
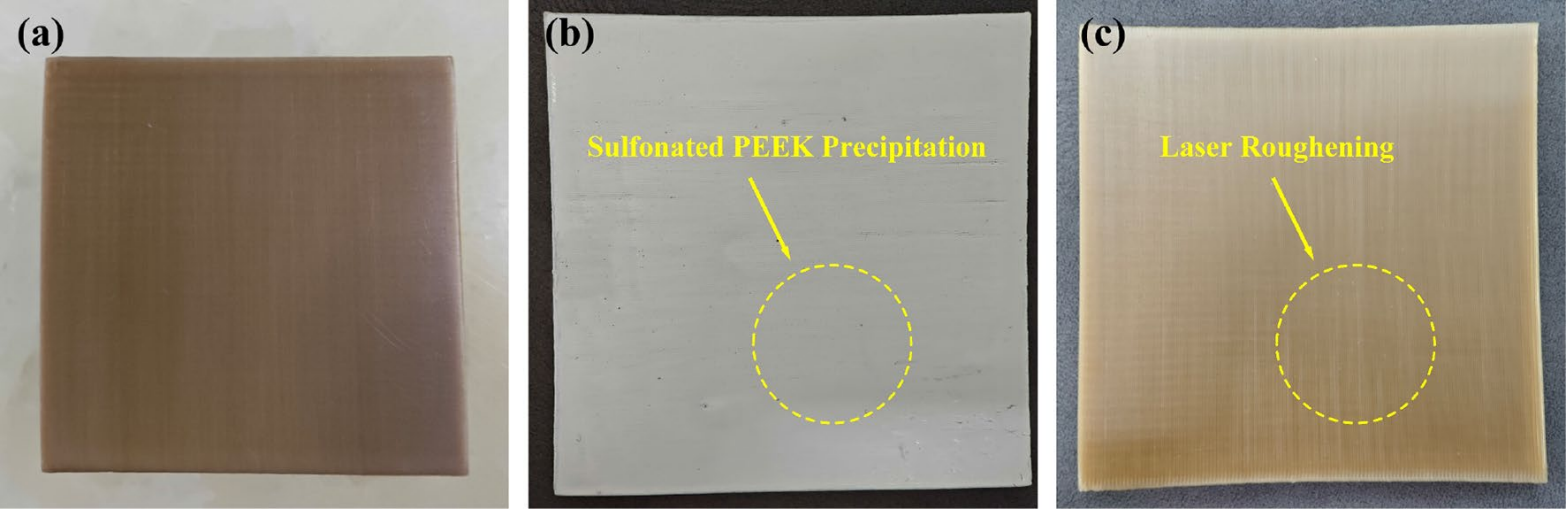
Display of PEEK sample surface modifications for: (**a**) SP-PEEK; (**b**) SPEEK120; (**c**) UV-PEEK15.

**Figure 6:**
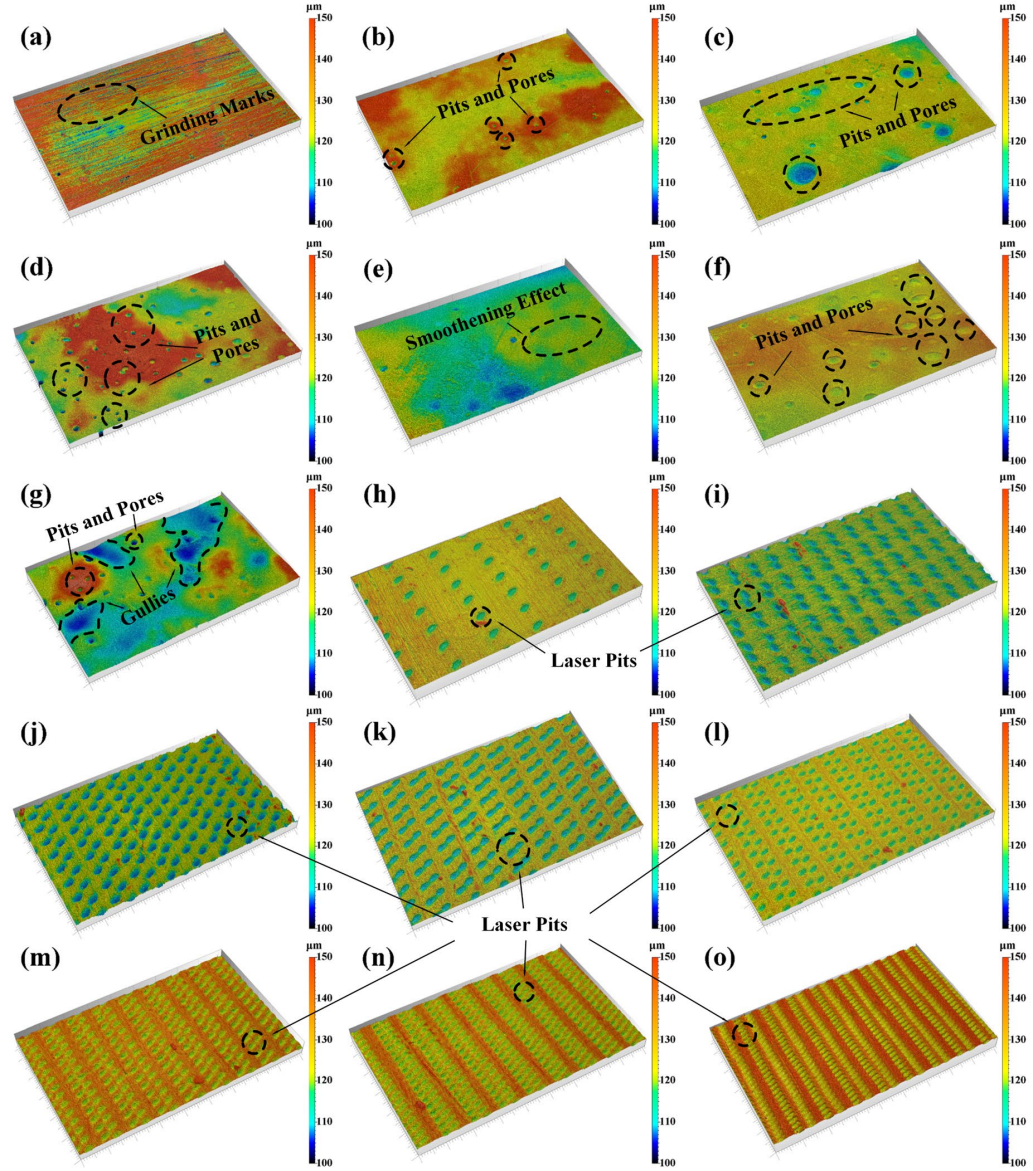
3D surface topography for: (**a**) SP-PEEK; (**b**) SPEEK30; (**c**) SPEEK60; (**d**) SPEEK120; (**e**) SPEEK180; (**f**) SPEEK240; (**g**) SPEEK300; (**h**) UV-PEEK27; (**i**) UV-PEEK24; (**j**) UV-PEEK21; (**k**) UV-PEEK18; (**l**) UV-PEEK15; (**m**) UV-PEEK12; (**n**) UV-PEEK9; and (**o**) UV-PEEK6. Swatch size: 0.919 mm × 0.575 mm.

**Figure 7:**
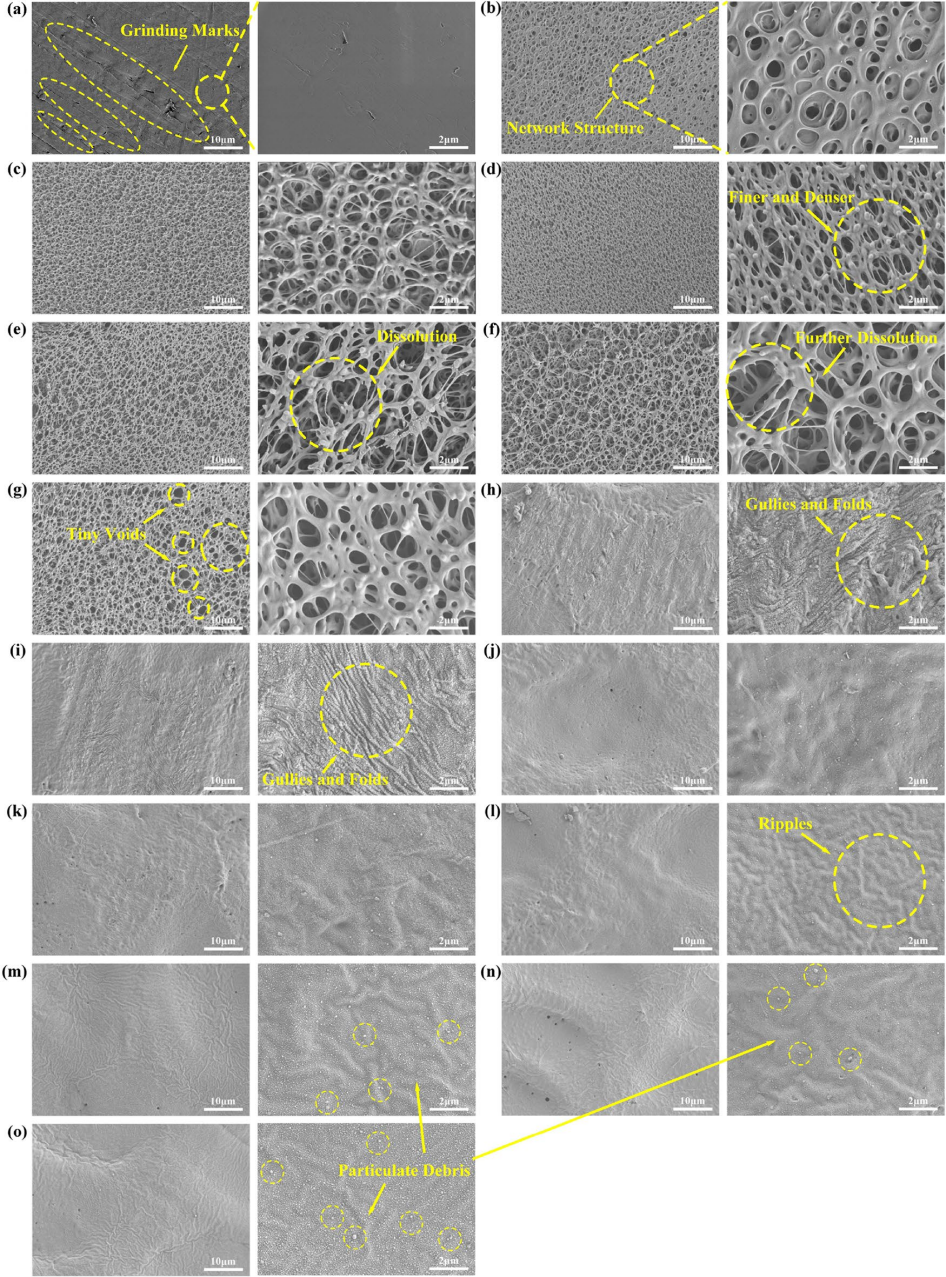
Surface micromorphology at 2000x and 10,000x magnifications for: (**a**) SP-PEEK; (**b**) SPEEK30; (**c**) SPEEK60; (**d**) SPEEK120; (**e**) SPEEK180; (**f**) SPEEK240; (**g**) SPEEK300; (**h**) UV-PEEK27; (**i**) UV-PEEK24; (**j**) UV-PEEK21; (**k**) UV-PEEK18; (**l**) UV-PEEK15; (**m**) UV-PEEK12; (**n**) UV-PEEK9; and (**o**) UV-PEEK6.

The surface morphology of PEEK materials underwent significant transformations after being subjected to various treatments, including concentrated sulfuric acid and ultraviolet laser treatments. These treatments altered the surface texture and significantly impacted the surface roughness, as comprehensively depicted in Figs. 6 and 7. While the polishing technique introduced discernible grinding marks onto the PEEK material’s surface (Fig. 6a), it also resulted in a remarkably smooth surface (Fig. 5a), as evidenced by the notably low surface roughness of 0.20 μm, indicating a highly polished texture. Notably, the treatment with concentrated sulfuric acid led to the precipitation of white sulfonated PEEK on the material’s surface (Fig. 5b). In the UV laser group, the laser’s roughening effect on the surface was also apparent (Fig. 5c).

During the initial phase of concentrated sulfuric acid treatment, the PEEK samples underwent marked changes in their macro and micro morphology. By the SPEEK30 stage, which entailed just 30 s of acid exposure, the surface displayed the emergence of irregular pits and pores (Fig. 6b), elevating the surface roughness to 0.59 μm. This trend intensified as the treatment advanced to SPEEK60, with the roughness reaching 0.80 μm, a testament to the potent effects of the acid. When the process advanced to SPEEK120, the surface roughness peaked at 1.15 μm, with an increase and deepening of pits and pores (Fig. 6d). Microscopic examinations revealed a distinct three-dimensional network structure that became more pronounced, finer, and denser as the treatment duration increased (Fig. 7d). However, the narrative shifted with SPEEK180. The previously rising surface roughness unexpectedly declined to 0.78 μm, suggesting a temporary smoothening effect imparted by the acid (Fig. 6e). Concurrently, microscopic observations unveiled signs of dissolution and degradation of the established three-dimensional network (Fig. 7e). This smoothening phase was short-lived. As the treatment extended to SPEEK240 and SPEEK300, the surface roughness values registered at 0.81 μm and 1.75 μm, respectively, hinting at a return to its initial rugged state. The surface was again characterized by irregular pits, pores, and gullies (Fig. 6g). On the microscopic front, the previously prominent three-dimensional network structure showed signs of further dissolution (Fig. 7f), and even some areas of the surface network structure completely dissolved to form tiny voids (Fig. 7g), transitioning into a less defined and more irregular pattern.

In contrast, ultraviolet laser treatment revealed a clear correlation between laser speed and surface morphology. Laser speed was identified as the key variable, and its modulation revealed a distinct trend in surface property transformations. Upon exposure to the highest laser speed of 2700 mm/s, as seen in the UV-PEEK27 sample, the surface roughness increased to 0.44 μm. A clear observation at this stage was the regularly distributed laser pits on the material's surface, each delving to a depth of 4.23 μm (Fig. 6h). As the laser speed was systematically decreased, marked transformations were evident on the surface. The roughness values began to rise, the pits deepened, and their distribution became denser. A declining laser speed intensified these surface alterations: increasing roughness, deepening pits, and a denser pit distribution. This trend culminated with UV-PEEK6, at a laser speed of 600 mm/s, where the surface roughness reached its zenith at 2.00 μm and pit depths extended to 5.73 μm. This behavior can be attributed to the prolonged duration of laser-material interaction, facilitating enhanced laser energy absorption by the material. This amplified energy exchange manifested as deeper pits and a more clustered distribution. Microscopically, these effects were even more conspicuous. The early phases, particularly within UV-PEEK27 and UV-PEEK24, were marked by the appearance of irregular gullies and folds (Fig. 7h,i), which subsequently became shallower by UV-PEEK21. Progressing to UV-PEEK15, these gullies and folds transitioned into subtle surface ripples (Fig. 7l). As the laser speed was further reduced, these textures became less prominent. Contrastingly, the particulate debris—a byproduct of the laser treatment—began to densely populate the material’s surface (Fig. 7o), with its features becoming more sharply defined.

**Figure 8:**
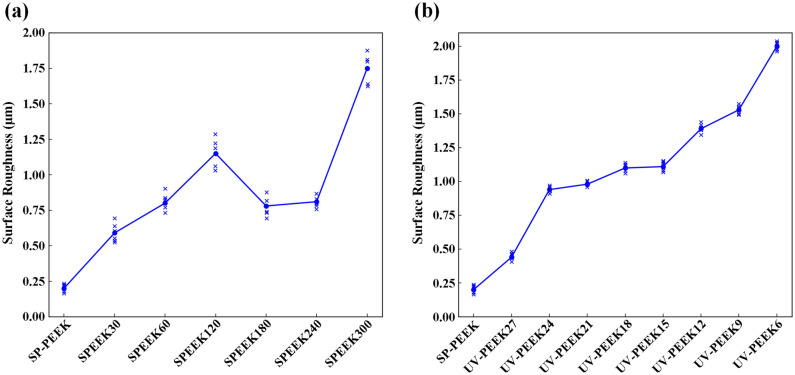
Surface roughness for: (**a**) concentrated sulfuric acid treatment group; (**b**) UV laser treatment group.

**Figure 9:**
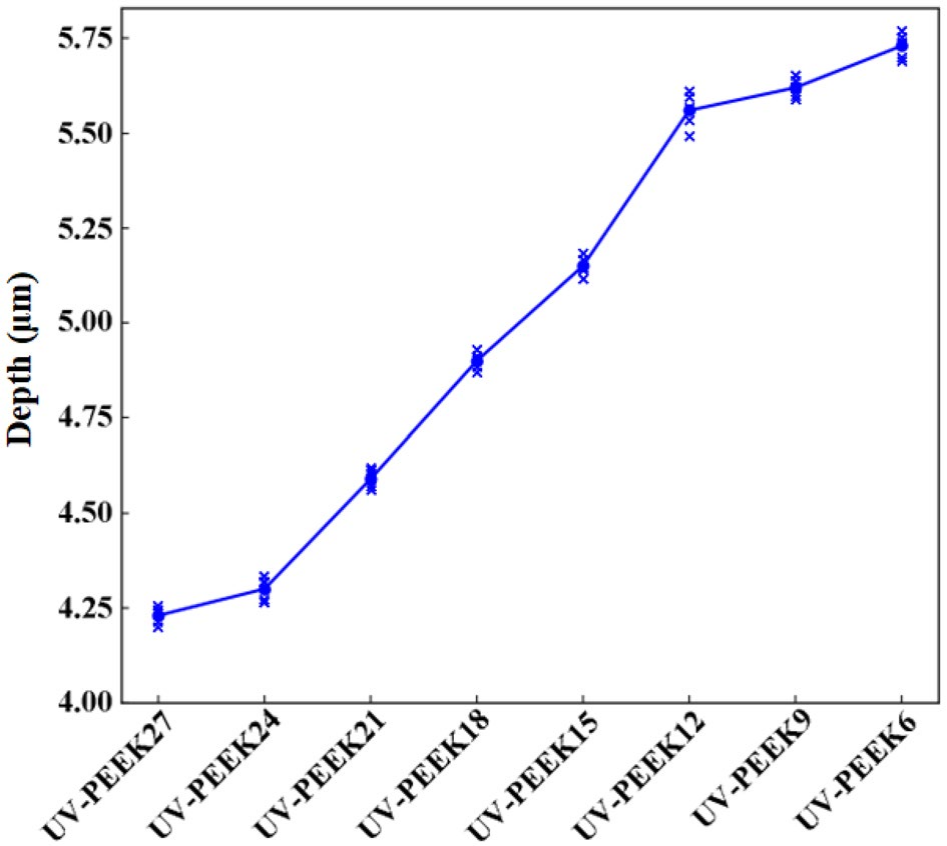
Pit depths for UV laser treatment group.

The alteration in roughness—initially increasing, then decreasing, and subsequently increasing again (as demonstrated in Fig. 8a)—in the concentrated sulfuric acid treatment group is closely intertwined with the formation, maturation, and eventual dissolution of the surface’s three-dimensional network structure. This process is consistent with previous research findings documenting a network structure’s emergence and increased surface complexity following acid treatment^26,27^. Moreover, the observed fluctuations in roughness resonate with the reported effects of acid treatments on surface roughness, highlighting the variability in surface morphology induced by such treatments^43^. As the treatment duration extends, increasingly unpredictable morphological alterations manifest on the material’s surface, including the emergence of irregular pits, pores, and gullies. This complexity in surface morphology reflects the unique interaction between the sulfuric acid and the PEEK material, resulting in a varied and irregular topography that defies simple quantification due to its random nature in size and depth. Conversely, the ultraviolet laser treatment group exhibits a direct correlation between laser speed and surface roughness (Fig. 8b), where modulating the laser speed alters the density and depth of the laser-induced pits (Fig. 9), showcasing higher controllability. Unlike the microscopic three-dimensional network structure invoked by the chemical method, the physical method displays a clear transition in surface texture as the laser speed varies—from irregular gullies to subtle ripples, and eventually to distinct particulate debris. This resulting texture, characterized by patterns of small mounds on a nanometer scale, introduces an additional surface roughness^44^. Both ways markedly alter PEEK’s surface morphology and roughness, which are pivotal in enhancing the material's bonding properties.

### Surface chemical composition

**Figure 10:**
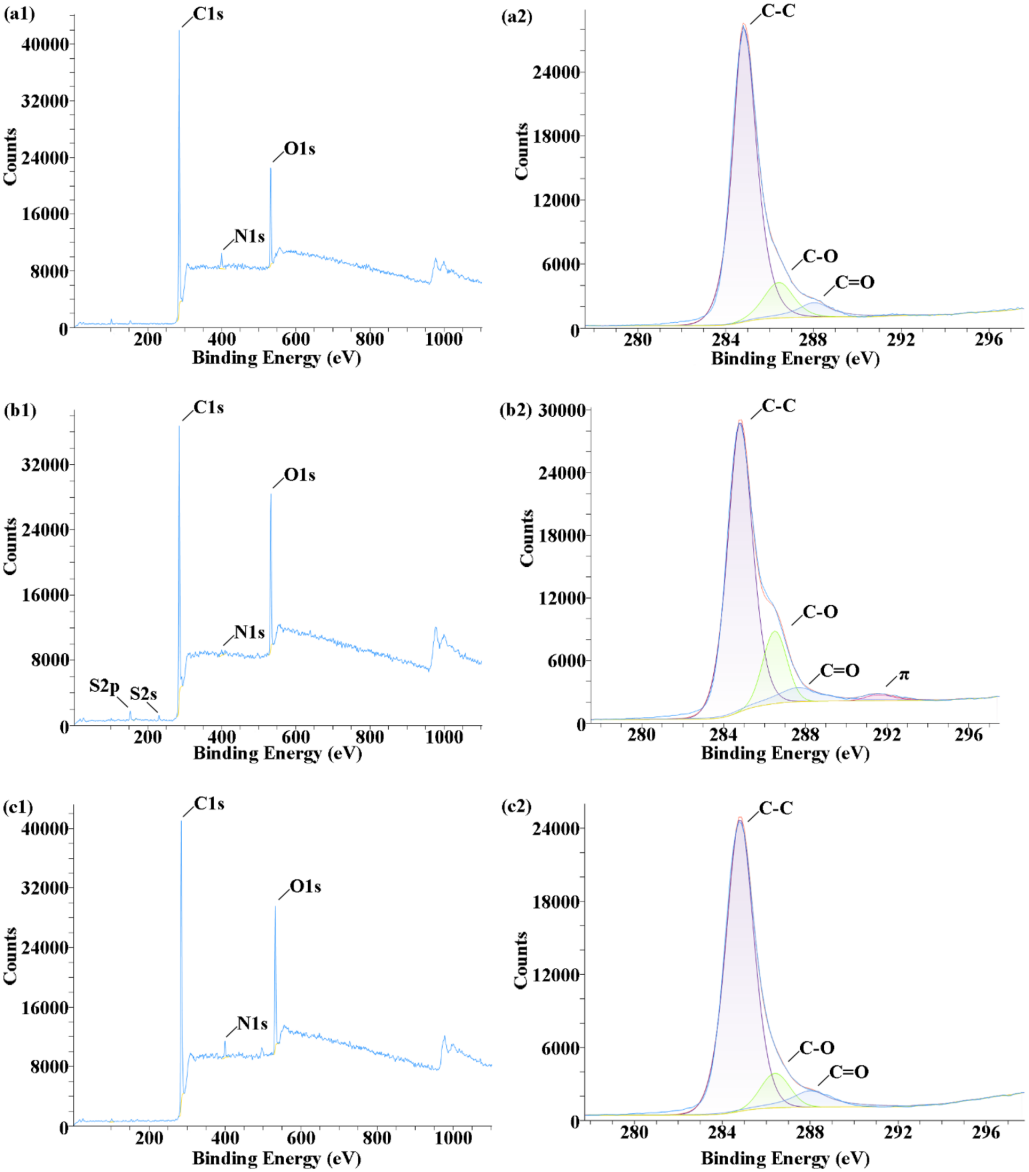
XPS full spectra (left) and C1s spectra (right) for: (**a**) SP-PEEK; (**b**) SPEEK120; (**c**) UV-PEEK15.

The two treatments induced significant alterations in the elemental and functional group composition on the PEEK surface, as revealed by XPS examination. SP-PEEK, SPEEK120, and UV-PEEK15 serve as representative samples, illustrating the variations in surface composition resulting from different treatments. In the XPS full spectrum, characteristic peaks for C1s (at 284.80 eV), O1s (at 532.12 eV), and N1s (at 401.64 eV) are discernible. Notably, for SPEEK120, additional peaks corresponding to S2p (at 168.91 eV) and S2s (at 232.56 eV) are evident, affirming the incorporation of sulfur following acid treatment. Both SPEEK120 and UV-PEEK15 exhibit an enhanced O1s peak intensity, indicative of a heightened oxygen presence on the surface, potentially resulting from oxidation. Within the C1s spectra, peaks are identified at 284.80 eV (C–C/C–H), 286.30 eV (C–O), and 287.60 eV (C=O). Figure 10 presents the XPS full spectra and C1s spectra for SP-PEEK, SPEEK120, and UV-PEEK15, showcasing the specific surface composition changes and the effects of the treatments on these representative samples.

**Figure 11:**
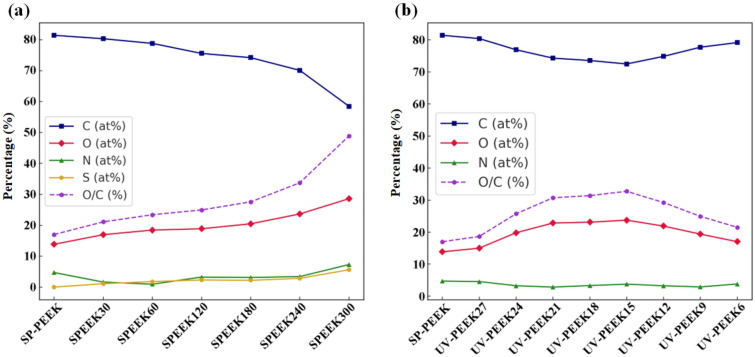
Surface elemental atomic percentage and O/C ratio for: (**a**) concentrated sulfuric acid treatment group; (**b**) UV laser treatment group.

The changes in surface elemental composition reflect the significant impact of concentrated sulfuric acid on the PEEK samples (Fig. 11a). For the SP-PEEK baseline, the carbon content was predominantly 81.44 at%. As the treatment duration increased, there was a noticeable reduction in carbon content. By SPEEK300, this value had decreased to 58.50 at%, marking a decline of nearly 23%. Concurrently, the oxygen content, initially 13.85 at% for SP-PEEK, consistently rose across the treated samples, peaking at 28.60 at% in SPEEK300. This upward trend indicates enhanced oxidation of the PEEK surface, likely resulting from its exposure to sulfuric acid and the subsequent development of oxygen-rich functional groups. While sulfur content was absent in SP-PEEK, it began to appear following the acid treatment, suggesting the sulfonation of the PEEK material. This content started at 1.09 at% in SPEEK30 and gradually escalated to 5.60 at% in SPEEK300, indicating the increasing degree of the material's sulfonation reaction as the acid treatment persisted. The O/C ratio, a valuable parameter for assessing oxidation levels, began at 17.01% for SP-PEEK. With prolonged acid exposure, this ratio consistently increased, reaching a significant 48.89% for SPEEK300. This marked rise emphasizes the extensive oxidative changes experienced by the PEEK surface due to the acid treatment. Figure 11b then illustrates the changes under UV laser treatment. Beginning with the highest speed at UV-PEEK27 (2700 mm/s), the carbon content slightly decreased to 80.43 at%, while the oxygen content edged up to 15.00 at%, resulting in an O/C ratio of 18.65%. Even at this swift laser speed, minor oxidative events or photochemical reactions induced by the laser appear to introduce subtle oxygen functionalities. The chemical shifts became more pronounced with a reduced laser speed, implying a longer laser-material interaction duration. At UV-PEEK24, the carbon content dropped to 76.93 at%, and the oxygen content increased to 19.81 at%. The O/C ratio climbed to 25.74%. This upward trend in oxidation was further evident at UV-PEEK15, manifesting a carbon content of 72.50 at% and an oxygen content of 23.74 at%, with the O/C ratio reaching 32.75%. These values reflect the laser’s enhanced photo-oxidative influence at slower speeds. Intriguingly, for UV-PEEK12, the trend slightly reversed, with the carbon content rising to 74.86 at% and oxygen content decreasing to 21.89 at%. The O/C ratio adjusted down to 29.25%. This deviation hints that, beyond certain processing conditions, the laser might cause a blend of both ablation and oxidation. Some oxidized surfaces might be removed, accounting for this observed fluctuation.

**Figure 12:**
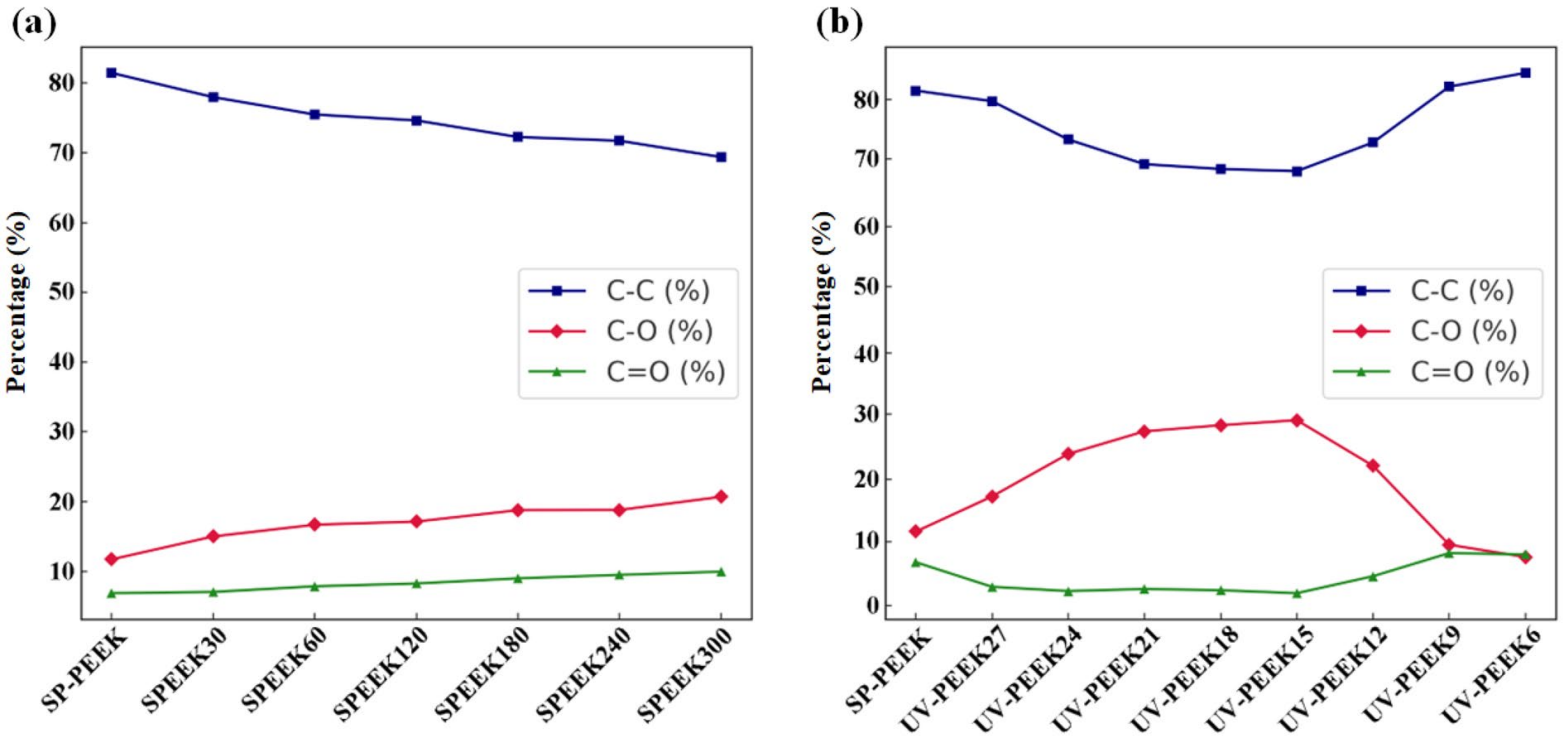
Major functional groups in C1s spectrum for: (**a**) concentrated sulfuric acid treatment group; (**b**) UV laser treatment group.

Shifting the focus to the main functional groups within the C1s peak spectrum (Fig. 12). The SP-PEEK sample showcased a dominant C–C bond content of 81.45%. With progressive sulfuric acid treatment, there was a steady decline in C–C content, dropping to 69.39% by SPEEK300. This reduction implies potential alterations or reactions in the polymer chain due to acid interactions. Simultaneously, there was a marked increase in the percentage of C–O, indicative of ether linkages or potential oxidation sites. Originating at 11.7% in SP-PEEK, the C–O content surged to 20.67% in SPEEK300. The content of the carbonyl (C=O) functional group also followed an ascending pattern, starting at 6.85% in SP-PEEK and rising steadily to 9.94% in SPEEK300. The emerging and expanding carbonyl groups indicate heightened oxidation of the polymer surface, potentially giving rise to ketones, esters, or carboxylic acid groups. Such growth in the C–O and C=O contents suggests that the acid treatment might catalyze the oxidation of polymer chains, leading to the emergence or amplification of oxygen-rich functionalities. The content of the carbonyl (C=O) functional group also demonstrated an ascending pattern. Starting at 6.85% in SP-PEEK, it steadily rose to 9.94% in SPEEK300. The emerging and growing carbonyl groups indicate heightened oxidation of the polymer surface, potentially leading to the generation of ketones, esters, or carboxylic acid groups. This suggests that acid treatment might catalyze the oxidation of polymer chains, resulting in the appearance or amplification of carbonyl-based functional groups. Regarding the UV laser treatment group, the C–C bond content in the UV-PEEK27 sample exhibited a notable reduction to 79.78%. Concurrently, there was a noteworthy increase in the C–O content, reaching 17.26%, while the C=O content diminished to 2.96%. This trend insinuates that, at this laser speed, ether linkages (C–O) are primarily formed, overshadowing the carbonyl (C=O) functionalities. As the laser speed decreased to UV-PEEK24, the C–C content receded to 73.72%. The C–O content surged to 23.99%, emphasizing the dominance of ether linkages. This pattern was even more accentuated for UV-PEEK15, where the C–C content plummeted to 68.72%, and the C–O soared to 29.33%. The C=O content reached its nadir at 1.95%. UV-PEEK12 brought an unexpected turn, with the C–C content recovering to 73.25%, the C–O content retracting to 22.11%, and a surge in the C=O content to 4.64%. This suggests a mixed influence of the laser, promoting both oxidation and ablation, and potentially influencing the formation and removal of specific functional groups. UV-PEEK9 and UV-PEEK6 further deviated from the trend. While the C–C content substantially increased, the C–O content showed a decline. Concurrently, the C=O content amplified, especially for UV-PEEK9 and UV-PEEK6, marking 8.3% and 8.08% respectively. This shift suggests the laser’s altered impact, highlighting carbonyl functionalities over ether linkages at these specific speeds.

The concentrated sulfuric acid treatment and UV laser treatment distinctly impacted the surface chemistry of PEEK samples. The sulfuric acid treatment consistently led to decreased carbon content, increased oxygen and sulfur levels, and a steady escalation in the O/C ratio, reflecting the material's intensified oxidation in the acidic milieu. This approach showcased a linear transformation, indicative of the acid's continuous and transformative effect on PEEK. The increase in the O/C ratio validates the augmented surface oxidation and functionalization, a pattern echoed in additional research focused on the impact of sulfonation^45,46^. Changes in functional group concentrations, including the rise in C–C and the reduction in C–O, lend further evidence to the chemical transformations prompted by sulfuric acid treatment^47^. While previous studies have documented the effects of UV laser treatment on surface elemental composition, it turns out that these effects exhibit a fluctuating pattern, especially in terms of the O/C ratio and the specific contents of C–C, C–O, and C=O. The laser speed played a pivotal role in these oscillations, highlighting the nuanced balance between oxidation, ablation, and potential bond reformation. These fluctuations, especially in the O/C ratio and functional group contents, emphasized the intricate and variable modifications the laser process can induce on the PEEK surface.

### Surface wettability and free energy

**Figure 13:**
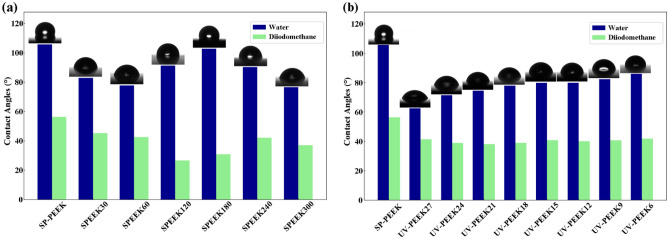
Contact angles with accompanying images (water) for: (**a**) concentrated sulfuric acid treatment group; (**b**) UV laser treatment group.

**Figure 14:**
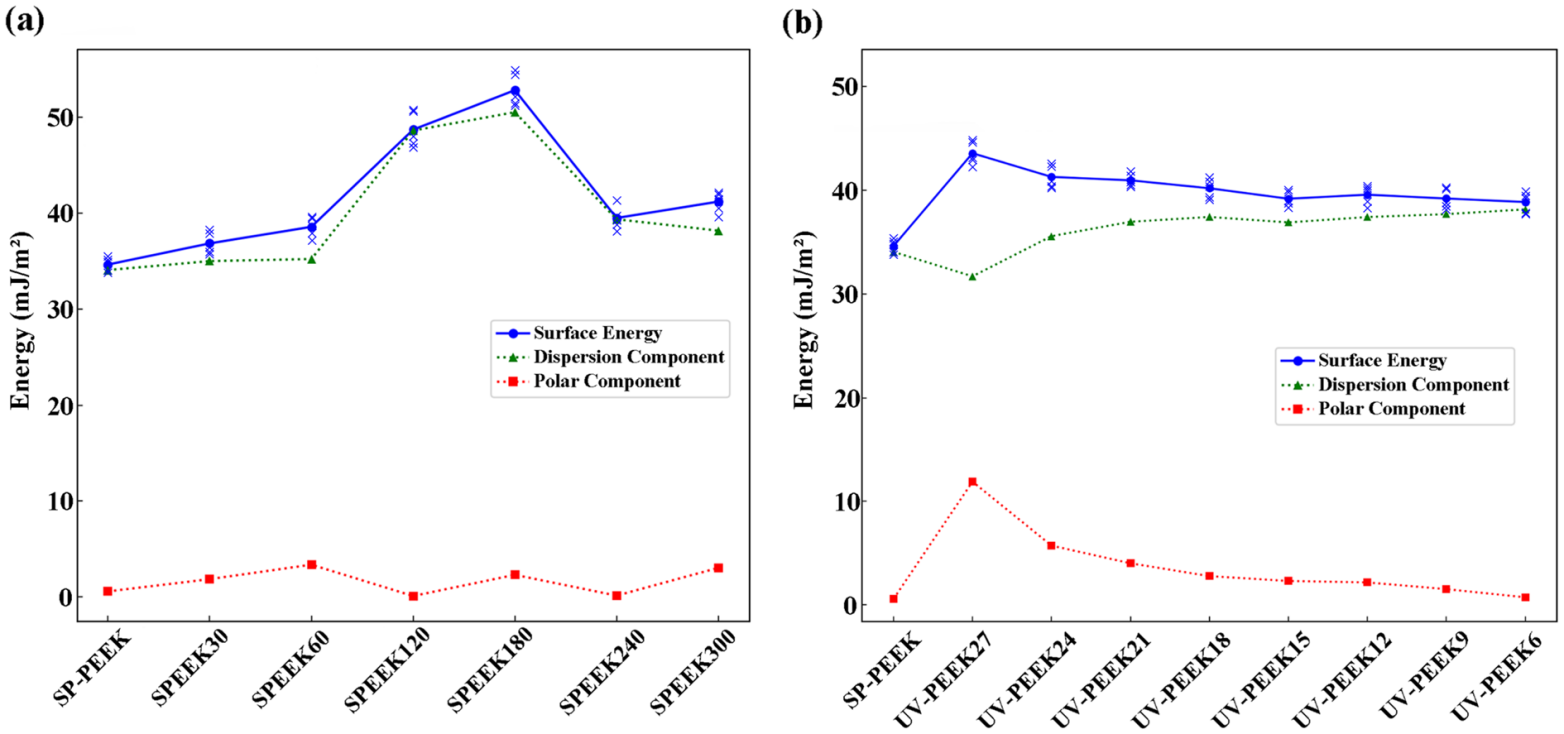
SFE for: (**a**) concentrated sulfuric acid treatment group; (**b**) UV laser treatment group.

The SP-PEEK distinctly demonstrates hydrophobicity, with a contact angle measurement of 105.78°. This behavior can be attributed to the PEEK polymer’s inherent nature and the specific surface morphology derived from its processing. The concentrated sulfuric acid treatment induces fluctuations in both contact angle, as depicted in Fig. 13a, and SFE, as detailed in Fig. 14a. With the introduction of the SPEEK30 sample, there was a notable decline in the contact angle to 82.90°, suggesting an increased hydrophilic tendency. This shift in wettability can be attributed to the influence of concentrated sulfuric acid, which likely induces etching effects, forming microstructures that enhance wettability. The trend, does not follow a linear trajectory. While the SPEEK60 sample further reduces to 77.75°, emphasizing its hydrophilic nature, SPEEK120 unexpectedly rises to 91.28°. A more pronounced increment to 102.81° in the SPEEK180 sample signifies a resurgence of hydrophobic characteristics. This fluctuation could be ascribed to the dual influence of the acid, introducing new functional groups and causing unique alterations in the surface morphology. Yet, when the samples reach SPEEK240 and SPEEK300, with contact angles of 90.23° and 76.61° respectively, the balance between chemical modifications and increased surface roughness appears to favor hydrophilicity. The evolution of SFE provides further insights. SP-PEEK commences with an SFE value of 34.64 mJ/m^2^. Progressing to SPEEK30 and SPEEK60, there’s a subtle ascent to 36.85 mJ/m^2^ and 38.58 mJ/m^2^, respectively. A more pronounced surge is evident in SPEEK180, reaching 52.84 mJ/m^2^. This increase in SFE might be linked to the rise of the polar component, suggesting a shift towards a more polar surface owing to the exposure or introduction of new functional groups. Nevertheless, post-SPEEK180, the trend exhibits some inconsistency. SPEEK240 and SPEEK300 register diminished measures, anchoring at 39.5 mJ/m^2^ and 41.21 mJ/m^2^, respectively. Despite this slight regression, these values remain considerably higher than the original SP-PEEK, highlighting the lasting effects of the acid treatment.

A marked transformation in contact angle is evident upon applying UV laser treatment (Fig. 13b), particularly at the rapid speed of 2700 mm/s (UV-PEEK27). The contact angle sharply descends to 62.55°, indicating a trend towards hydrophilicity. This shift is not merely superficial; it stems directly from the UV laser’s capacity to modify the surface attributes. At the microscopic level, laser treatment promotes surface roughening, potentially amplifying the surface’s interaction area with water. Concurrently, at the molecular level, UV radiation may be instrumental in introducing or modifying hydrophilic functional groups, thereby enhancing the surface’s affinity for water. However, this pronounced hydrophilicity does not remain constant. With extended UV treatment durations, represented by decreasing speeds from UV-PEEK24 to UV-PEEK6, the contact angle begins its upward climb, settling at 86.03° at 600 mm/s. This re-emergence of hydrophobicity suggests complex surface dynamics. Prolonged UV exposure might instigate specific chemical alterations, such as polymer degradation or the formation of cross-linked networks, which counteract the initial hydrophilic advancements. The variations in SFE provide a more nuanced understanding of a material’s surface, as illustrated in Fig. 14b. Initially, SP-PEEK registers an SFE of 34.64 mJ/m^2^. With the integration of UV treatment in UV-PEEK27, there’s a significant rise to 43.61 mJ/m^2^. This increase aligns with the contact angle observations, emphasizing the enhanced hydrophilicity achieved in the initial UV treatment. Yet, reflecting the patterns observed in contact angles, the SFE values progressively decline from UV-PEEK24 to UV-PEEK6. The decrease from 41.32 to 38.89 mJ/m^2^ illustrates a surface becoming less conducive to wetting. This pattern infers that while UV treatment can initially elevate SFE, extended exposure might introduce modifications that offset these gains, underscoring the delicate interplay of factors influencing surface properties.

These two treatments have conclusively demonstrated their effectiveness in enhancing hydrophilicity and improving the SFE of PEEK samples. The effects of concentrated sulfuric acid treatment on the surface properties of PEEK samples displayed a non-linear trend, transforming the initially hydrophobic SP-PEEK to a more hydrophilic surface, but with variations observed as the treatment progressed. The oscillation in contact angles reflects the dual impact of the acid treatment on surface morphology and chemical modifications. Such fluctuations echo observations in varied studies, where the effect of sulfonation on hydrophilicity was found to oscillate due to differing degrees of surface modification^27,31^. Upon examining relevant studies, there is a noted disparity in findings regarding whether sulfonation treatment increases hydrophilicity^32,48,49^ or hydrophobicity^26,29^. This variation highlights the intricate effects of sulfonation conditions on material surface characteristics, indicating that both enhancements and reductions in hydrophilicity or hydrophobicity can be traced back to the fluctuations caused by the sulfonation process. SFE mirrored this non-linear trend, starting with a modest increase before exhibiting inconsistencies, albeit ending on a higher note than SP-PEEK. On the other hand, UV laser treatment, particularly at higher speeds, initially heightened hydrophilicity as evidenced by a sharp reduction in contact angles. However, with prolonged exposure at slower speeds, a resurgence of hydrophobicity was observed. Such a transition towards increased hydrophobicity aligns with observations from other studies, which have documented an increase in the contact angle following laser treatment, suggesting an enhancement in hydrophobic properties^50^. This alignment with the Cassie wetting theory further underscores the role of laser-induced surface modifications in manipulating material wettability^51^. The SFE, aligning with the hydrophilicity trend, gradually diminished with prolonged UV exposure at slower speeds.

### Surface binding force

**Figure 15:**
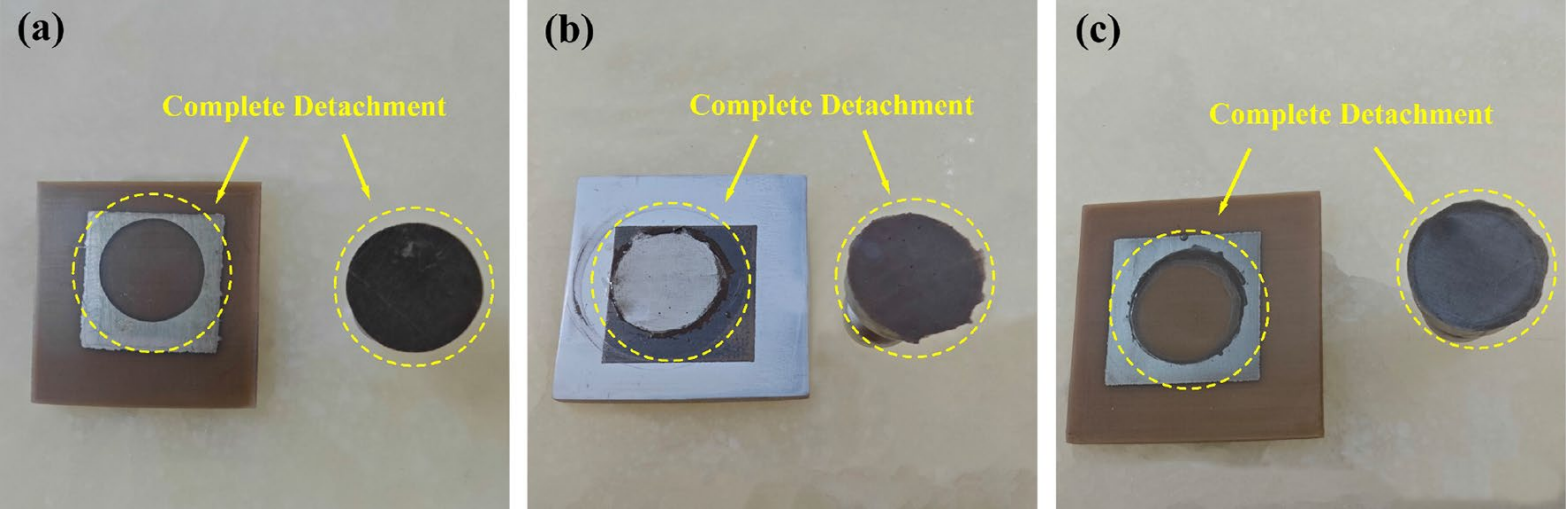
Demonstration of complete detachment for: (**a**) SP-PEEK; (**b**) SPEEK120; (**c**) UV-PEEK15.

The treated PEEK samples demonstrated significant improvements in binding force compared to the control group (SP-PEEK), which exhibited a minimal binding force of 0.37 MPa. Among the treated groups, SPEEK120 and UV-PEEK15 stood out with their notably enhanced binding forces, measuring 1.99 MPa and 2.21 MPa, respectively. The results of the binding force are presented in Table 1. The efficacy of the pull-off method in enabling the complete detachment of the nanoparticle silver ink layer from the PEEK substrate was robustly demonstrated across experiments (Fig. 15).

**Table 1:** Binding force.

Sample	Binding force (MPa)
SP-PEEK	0.37
SPEEK30	1.15
SPEEK60	1.76
SPEEK120	1.99
SPEEK180	0.89
SPEEK240	1.15
SPEEK300	1.49
UV-PEEK27	1.27
UV-PEEK24	1.58
UV-PEEK21	1.79
UV-PEEK18	1.84
UV-PEEK15	2.21
UV-PEEK12	1.87
UV-PEEK9	1.55
UV-PEEK6	1.09
LS-PEEK	2.77
SL-PEEK	1.78

**Figure 16:**
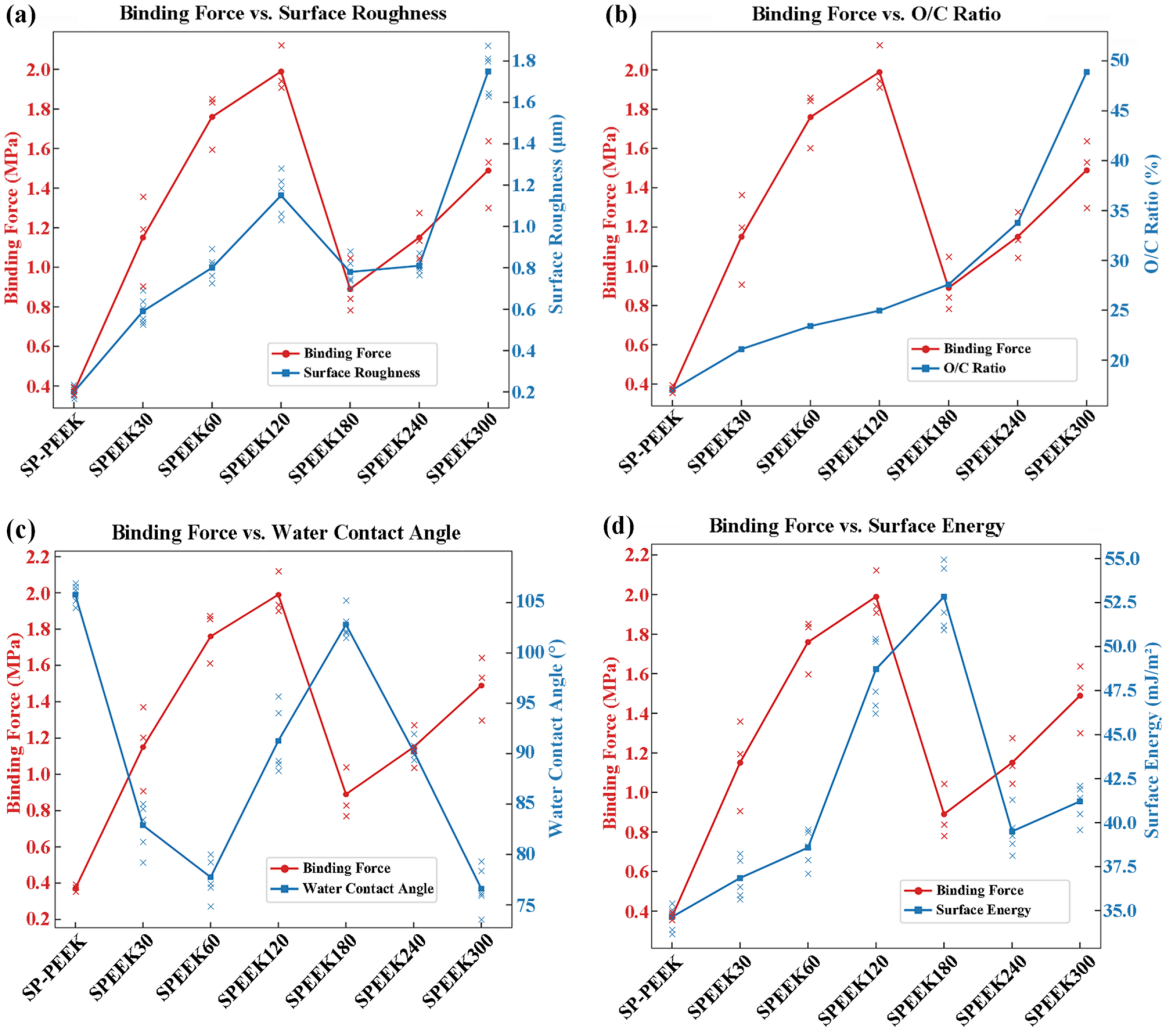
Correlations and trends in binding force for SPEEK samples with: (**a**) Surface roughness; (**b**) O/C ratio; (**c**) Water contact angle; (**d**) SFE.

For the concentrated sulfuric acid treatment group, surface roughness prominently dictates the binding force between the nanoparticle silver ink layer and the treated surface. This relationship is particularly pronounced during the initial and intermediate treatment stages. The initial condition, represented by SP-PEEK, establishes a baseline where low surface roughness (0.20 μm) aligns with minimal binding force (0.37 MPa). With treatment progression, as exemplified by the SPEEK30 and SPEEK60 samples, both surface roughness and binding force exhibit a synchronized increase. This consistent rise underscores the principle that increased roughness augments mechanical interlock, strengthening the bond between the nanoparticle ink layer and the substrate. SPEEK120 symbolizes an intermediary phase, with roughness and binding force reaching their zenith at 1.15 μm and 1.99 MPa, respectively. However, this peak is followed by a decline in SPEEK180, suggesting there might be an optimal roughness threshold to achieve maximum binding force. Surpassing this threshold may present obstacles like material degradation or suboptimal surface characteristics that hinder effective bonding. Although SPEEK300’s roughness amplifies to a remarkable 1.75 μm, the corresponding increase in binding force is tempered, reaching 1.49 MPa. This nuanced trend indicates that factors other than roughness might influence the binding force in extended treatments. Further investigation into the correlation between binding force and other surface characteristics was conducted and is presented in Fig. 16. The binding force exhibits a moderate positive correlation with surface roughness, evidenced by a Pearson coefficient of r = 0.66. This trend underscores the principle that rougher surfaces typically offer better mechanical adhesion due to increased interlocking. Conversely, the relationship between binding force and water contact angle is moderately negative (r =  − 0.70), suggesting that surfaces with higher wettability, indicated by lower contact angles, are associated with stronger binding forces. While the O/C ratio and SFE also relate positively to binding force, with coefficients of r = 0.30, these correlations are relatively weak. These latter findings imply that, although there is a slight tendency for binding force to increase with higher O/C ratios and SFE, these factors do not have as substantial an impact as surface roughness or wettability. The collective analysis presents a multifaceted picture, with surface roughness and hydrophobicity emerging as the primary factors, but also hints at the nuanced influence of surface chemistry and energy on the adhesive performance of the treated materials.

**Figure 17:**
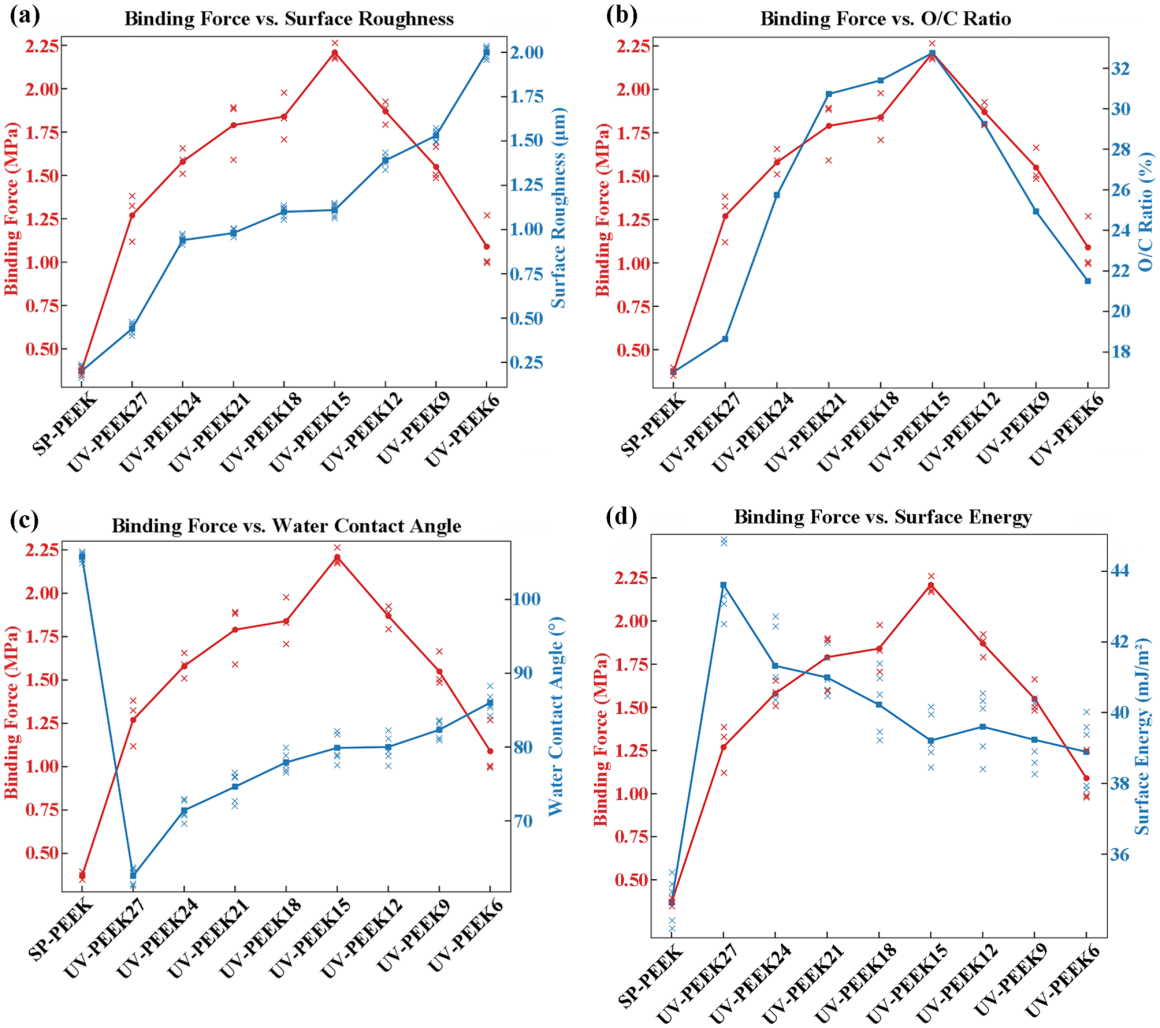
Correlations and trends in binding force for UV-PEEK samples with: (**a**) Surface roughness; (**b**) O/C ratio; (**c**) Water contact angle; (**d**) SFE.

The UV laser modification of PEEK surfaces reveals a pronounced interplay between the binding force and the surface’s elemental composition, especially epitomized by the O/C ratio. The intricacies of this relationship are further elucidated by examining the nuances of the C1s peak spectrum. From a binding force perspective, a discernible progression is observed. As the laser treatment advances from UV-PEEK27 to UV-PEEK15, there’s a systematic amplification in binding force. This crescendo peaks with UV-PEEK15, registering a binding force of 2.21 MPa. However, this zenith is followed by a decline, with UV-PEEK6 finally marking a binding force of 1.09 MPa. Shifting the focus to elemental composition, the O/C ratio—a barometer for surface oxidation—mirrors the binding force trajectory. Commencing at 17.01% for SP-PEEK, the O/C ratio ascends to its apogee of 32.75% for UV-PEEK15, only to recede afterward. This upward shift and subsequent decline suggest that an elevation in surface oxidation, manifested by a burgeoning presence of oxygen-containing functional groups, is pivotal for bolstering binding force. These oxygen-rich groups can enhance affinity and cohesion with the nanoparticle silver ink layer, solidifying the bond. The insights gleaned from the C1s peak spectrum further bolster this hypothesis. UV-PEEK15, boasting the apex in binding force, displays a diminished C–C content (68.72%) juxtaposed with an enriched C–O content (29.33%). Such a distribution underscores the prevalence of oxygen-rich functional groups, pivotal for binding enhancement. Deviating from UV-PEEK15, the C–C content swells with faster and slower laser speeds while the C–O content contracts. This shift, indicating a reduction in oxygen-rich groups, aligns seamlessly with the observed attenuation in binding forces. For UV laser-treated PEEK samples, Fig. 17 delineates the associations between binding force and surface attributes. Correlation analysis indicates a strong positive relationship between binding force and the O/C ratio (r = 0.92), underscoring the importance of surface oxidation in enhancing adhesive interactions. The heightened presence of oxygen-containing groups with increased O/C ratios bolsters the binding force, reaching an apex at UV-PEEK15. This is contrasted by a moderate negative correlation with the water contact angle (r =  − 0.60), implying that increased wettability favors stronger binding. Additionally, a moderate positive correlation with SFE (r = 0.52) suggests that surfaces with higher energy are more conducive to adhesion, affirming the role of SFE in the adhesion process. Meanwhile, the positive yet weaker correlation with surface roughness (r = 0.38) indicates its lesser, albeit significant, contribution to adhesive strength compared to the chemical and wettability characteristics of the UV-treated PEEK surfaces. These findings collectively elucidate the pivotal influences shaping adhesive performance, with chemical composition and surface wettability taking precedence over mere topographical features.

**Figure 18:**
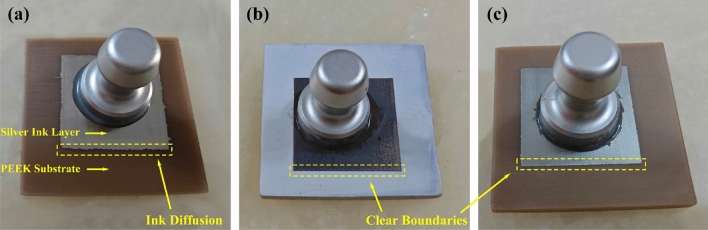
Boundary morphologies of nanoparticle silver ink layer for: (**a**) SP-PEEK; (**b**) SPEEK120; (**c**) UV-PEEK15.

**Figure 19:**
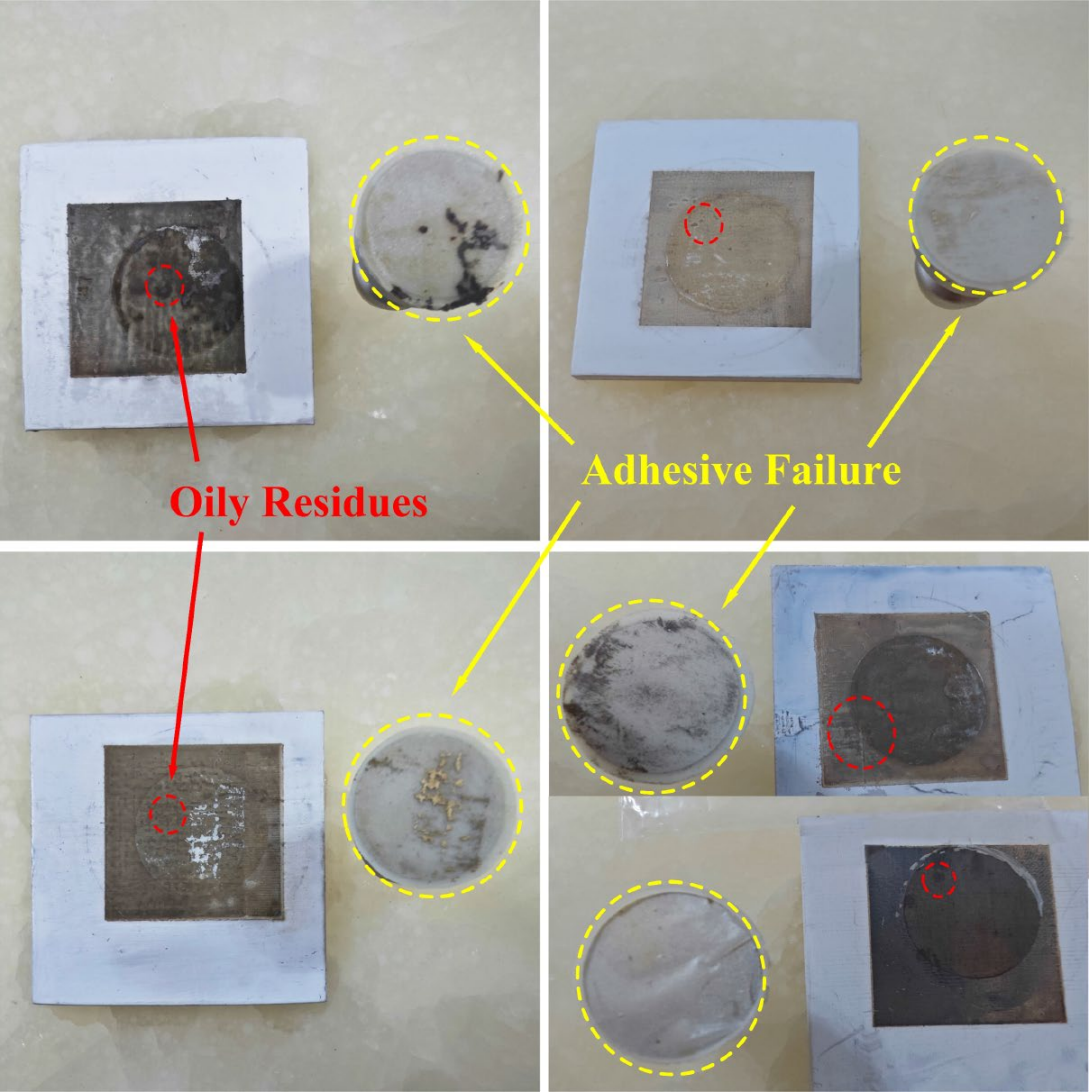
Adhesive failure: epoxy resin’s binding to nanoparticle silver ink layer.

In addition, further discoveries were made regarding the boundary interactions between the nanoparticle silver ink layer and the PEEK substrate. In the SP-PEEK group, noticeable ink diffusion was evident at the boundary of the silver layer. In contrast, the SPEEK and UV-PEEK groups displayed superior nanoparticle silver ink layer morphologies with clear, well-defined boundaries, highlighting the crucial role of surface modification in achieving a high-quality ink layer. These morphological distinctions are vividly illustrated in Fig. 18. Furthermore, the sample treated with sulfonation demonstrated additional complexity in manufacturing. The emergence of oily residues from the acid-altered PEEK was observed during the printing platform’s heating phase. This phenomenon led to adhesive failure, as the epoxy resin could not effectively bond to the silver layer, culminating in incomplete detachment as shown in Fig. 19. The extent of adhesive failure corresponded with the length of sulfonation treatment and the platform’s heating temperature. Sulfonation introduces polar sulfonic acid groups into PEEK, potentially destabilizing its molecular structure and promoting decomposition when exposed to high temperatures. The resulting oily residues, likely products of molecular changes and phase separation, implicate over-sulfonation in the degradation of adhesive qualities. Although sulfonation is intended to improve binding by increasing surface roughness and adding oxygen-rich groups, a critical limit exists. Overstepping this boundary can lead to a decline in adhesive effectiveness despite other surface properties continuing to improve. Precise calibration of the sulfonation process is therefore critical to enhance binding capabilities while avoiding the adverse effects of over-sulfonation and ensuring adhesive reliability.

### Exploration of cross-approach treatments

Building upon the previous experiments, this section explores the possibility of cross-approach treatments by combining the most effective individual methods, UV-PEEK15 and SPEEK120, to investigate potential synergistic effects on adhesion enhancement. Two sequential treatment groups are introduced: Laser-Sulfonated PEEK (LS-PEEK) for the group treated first with UV laser and then with sulfuric acid, and Sulfonated-Laser PEEK (SL-PEEK) for the group treated first with sulfuric acid and then with UV laser.

**Figure 20:**
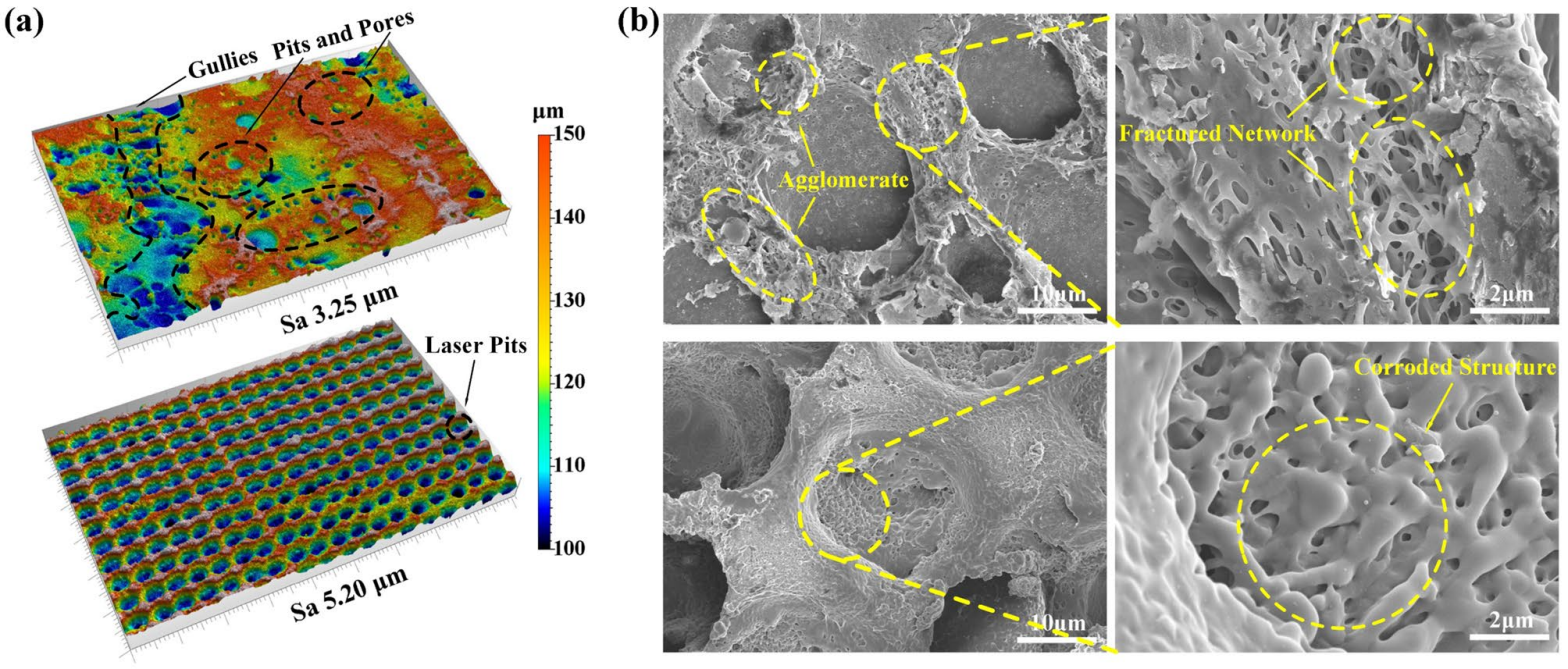
Surface morphology of LS-PEEK and SL-PEEK: (**a**) 3D Surface topography (Swatch size: 0.919 mm × 0.575 mm), featuring LS-PEEK in the upper part and SL-PEEK in the lower part. (**b**) Surface micromorphology at ×2000 and ×10,000 magnifications, showcasing LS-PEEK in the upper part and SL-PEEK in the lower part.

In the investigation of surface morphology, the surface roughness for LS-PEEK and SL-PEEK showed marked increases to 3.25 μm and 5.20 μm, respectively, indicating a substantial enhancement in surface roughness relative to SPEEK120 at 1.15 μm and UV-PEEK15 at 1.11 μm. Figure 20 visually depicts the impact differences of cross-approach treatments. Specifically, Fig. 20 a illustrates the 3D surface topography changes. For LS-PEEK, applying sulfuric acid to laser-treated surfaces intensified the sulfonation effect, transforming the previously ordered distribution of laser pits into a complex landscape populated with random pits, pores, and gullies, significantly altering the morphology beyond the original laser treatment effects. Conversely, SL-PEEK experienced an enhancement of the laser effect post-sulfonation, leading to the formation of deeper laser pits with an average depth of about 13.36 μm, thereby contributing to its higher surface roughness. Microscopic observations, as shown in Fig. 20b, revealed that in LS-PEEK, concentrated sulfuric acid intensely affected the areas around the laser pits, while the interior of the pits experienced only minimal sulfonation corrosion. This resulted in a morphology characterized by agglomerates of decomposed material and a fractured network, deviating from the typical fine three-dimensional network formed by sulfonation alone. In SL-PEEK, the laser treatment post-sulfonation erased the fine three-dimensional network structure, resulting in a relatively flat area around the laser pits, while a distinctly corroded three-dimensional network was observed inside the laser pits.

In evaluating the surface chemical composition post-treatment, LS-PEEK and SL-PEEK exhibit similar O/C ratios, with LS-PEEK at 26.05% and SL-PEEK at 23.97%, aligning closely with that of SPEEK120 (24.96%), as detailed in Table 2. This suggests that the sequential treatments maintain the oxygen content established by the sulfuric acid exposure. However, notable differences arise in the sulfur content and functional group distribution between the two sequential treatments. For LS-PEEK, undergoing UV laser treatment prior to sulfuric acid application results in a slight increase in surface sulfur content to 2.69 at%. This is because the surface after laser treatment increases the contact area with concentrated sulfuric acid, allowing for more sulfur incorporation. In contrast, SL-PEEK experiences a reduction in surface sulfur to 0.79 at% following the laser treatment post-acid exposure, suggesting that the laser treatment removes sulphonated PEEK from the surface. The analysis of major functional groups in the C1s spectrum (Table 3) reveals substantial shifts attributable to the order of treatments. LS-PEEK demonstrates a significant rise in C–O bonds to 50.58% alongside a reduction in C–C bonds to 46.53%, indicating enhanced oxidation and possibly increased hydrophilicity. This change suggests that the laser treatment following sulfuric acid application can further oxidize the surface, enriching it with oxygen-containing functional groups. The enhanced C–O formation in LS-PEEK could be advantageous for improving adhesion characteristics due to increased surface polarity and potential for better interaction with the nanoparticle silver ink. Conversely, SL-PEEK shows an increase in C–C bonds to 90.27% and a decrease in C-O bonds to 7.71% compared to LS-PEEK, pointing towards a potentially reduced hydrophilicity. The laser treatment destroyed the acid-treated surface structure and formed a high proportion of C–C, which may reduce the surface's ability to bond effectively with the nanoparticle silver ink.

**Table 2:** Surface elemental atomic percentage.

Sample	C (at%)	O (at%)	N (at%)	S (at%)	O/C (%)
SPEEK120	75.58	18.87	3.24	2.31	24.96
UV-PEEK15	72.50	23.74	3.76	N/A	32.75
LS-PEEK	75.09	19.56	2.66	2.69	26.05
SL-PEEK	77.52	18.58	3.11	0.79	23.97

**Table 3:** Major functional groups in C1s spectrum.

Sample	C–C (%)	C–O (%)	C=O (%)
SPEEK120	74.64	17.12	8.24
UV-PEEK15	68.72	29.33	1.95
LS-PEEK	46.53	50.58	2.89
SL-PEEK	90.27	7.71	2.02

The contact angle results, as detailed in Table 4, corroborate the conjectured changes in hydrophilicity inferred from the chemical composition analysis. LS-PEEK, with a water contact angle of 85.64°, displays moderately increased hydrophilicity compared to its individual treatment counterparts, likely due to its enhanced oxygen-containing functional groups. In contrast, SL-PEEK, which shows a higher water contact angle of 93.97°, appears to become more hydrophobic, aligning with the increase in C–C bonds and the decrease in oxygen-containing groups. The SFE outcomes, also presented in Table 4, further reflect these alterations. LS-PEEK exhibits an increased SFE of 49.60 mJ/m^2^, suggesting a surface more conducive to adhesion due to its balanced dispersive and polar components. Conversely, SL-PEEK’s SFE decreases to 36.31 mJ/m^2^, indicating a surface less favorable for adhesion, which is consistent with its higher hydrophobicity and reduced polar component. These SFE changes, combined with the water contact angle findings, validate the surface chemical composition results, illustrating a clear distinction between the effects of the two sequential treatment processes on the PEEK surface properties.

**Table 4:** Contact angle and surface free energy.

Sample	Water contact angle (°)	Diiodomethane contact angle (°)	Polar component (mJ/m^2^)	Dispersion component (mJ/m^2^)	Surface free energy (mJ/m^2^)
SPEEK120	91.28	26.66	0.09	48.63	48.72
UV-PEEK15	79.89	40.86	2.30	36.92	39.22
LS-PEEK	85.64	16.62	0.03	49.57	49.60
SL-PEEK	93.97	47.35	0.05	36.26	36.31

From the binding force results (Table 1), LS-PEEK demonstrated a binding force of 2.77 MPa, while SL-PEEK achieved a binding force of 1.78 MPa. These results clearly illustrate the significant impact of treatment sequence on binding force outcomes, with LS-PEEK showing markedly superior adhesion capabilities. This enhanced performance of LS-PEEK highlights the potential for optimizing adhesion properties through strategic sequencing of treatments. Comparing these results with those of SPEEK120 and UV-PEEK15 further underscores the effectiveness of cross-approach treatments. Such a comprehensive examination of binding forces across different treatment approaches is crucial for developing more reliable and durable PEEK-based components.

## Conclusion

This study illuminates the substantial impact of surface treatments on enhancing adhesion between 3D printed PEEK substrates and nanoparticle silver ink layers, marking a pivotal advancement for the additive manufacturing domain. Employing two prime surface modification techniques—concentrated sulfuric acid treatment and UV laser treatment—The present work identifies conditions that amplify the binding force, crucial for the durability of 3D printed components, and delves into the resulting physical and chemical transformations. It further explores how parameter variations drive mechanistic changes in the adhesion processes, highlighting the nuanced interplay between treatment methods and material responses. The notable findings are encapsulated as follows:

1. For concentrated sulfuric acid treatment, surface roughness emerged as a cardinal determinant of binding force. The peak was witnessed at SPEEK120 with an impressive binding force value of 1.99 MPa, signifying a 5.4-fold increase from SP-PEEK. However, over-processing manifested in potential drawbacks, such as the emergence of oily substances during heating, underscoring the need for balanced treatments.
2. For UV laser treatment, a pronounced correlation between binding force and surface elemental content, especially the O/C ratio, was observed. UV-PEEK15 emerged as a highlight, registering a binding force of 2.21 MPa, almost a sixfold increase from SP-PEEK. The enhancement in binding force was intimately tied to the rise in the O/C ratio and the presence of oxygen-containing functional groups.
3. Both treatments markedly enhanced the hydrophilicity, SFE, and the O/C ratio of PEEK, which translated into discernible improvements in the morphological quality of metal layers. These enhancements were integral in fostering better adhesion between the PEEK substrate and nanoparticle silver ink layer, facilitating the preparation of high-quality metal layers, a prerequisite in precision manufacturing. Additionally, the modifications notably ameliorated ink diffusion, a crucial determinant for achieving optimal metal layer quality, thereby underscoring the pivotal role of surface modifications in advancing the state of additive manufacturing applications.
4. The emergence of oily residues after sulfuric acid treatment, particularly during the heating stage of nanoparticle silver ink layer application, was identified as a potential challenge. This phenomenon underscored the application of acid-treated materials at high temperatures manifested a realm of uncertainty, necessitating further exploration in residue management to ensure consistent binding performance and material stability.
5. Cross-approach treatment effectiveness, as demonstrated by the binding force results from LS-PEEK (2.77 MPa) and SL-PEEK (1.78 MPa), reveals the critical influence of treatment sequence on adhesive outcomes. The superior performance of LS-PEEK underscores the potential of strategic treatment sequencing to optimize adhesion properties significantly. Furthermore, these results indicate that appropriate combination treatments can achieve better binding forces than individual treatments alone, offering a promising avenue for enhancing the durability and functionality of 3D printed electronics.

## Data Availability

All data generated or analyzed during this study are included in this published article.
